# An immunostimulatory dual-functional nanocarrier that improves cancer immunochemotherapy

**DOI:** 10.1038/ncomms13443

**Published:** 2016-11-07

**Authors:** Yichao Chen, Rui Xia, Yixian Huang, Wenchen Zhao, Jiang Li, Xiaolan Zhang, Pengcheng Wang, Raman Venkataramanan, Jie Fan, Wen Xie, Xiaochao Ma, Binfeng Lu, Song Li

**Affiliations:** 1 Center for Pharmacogenetics, School of Pharmacy, University of Pittsburgh, Pittsburgh, Pennsylvania 15261, USA; 2 Department of Pharmaceutical Sciences, School of Pharmacy, University of Pittsburgh, Pittsburgh, Pennsylvania 15261, USA; 3 University of Pittsburgh Cancer Institute, University of Pittsburgh, Pittsburgh, Pennsylvania 15261, USA; 4 Department of Immunology, School of Medicine, University of Pittsburgh, Pittsburgh, Pennsylvania 15261, USA; 5 Department of Surgery, School of Medicine, University of Pittsburgh, Pittsburgh, Pennsylvania 15261, USA

## Abstract

Immunochemotherapy combines a chemotherapeutic agent with an immune-modulating agent and represents an attractive approach to improve cancer therapy. However, the success of immunochemotherapy is hampered by the lack of a strategy to effectively co-deliver the two therapeutics to the tumours. Here we report the development of a dual-functional, immunostimulatory nanomicellar carrier that is based on a prodrug conjugate of PEG with NLG919, an indoleamine 2,3-dioxygenase (IDO) inhibitor currently used for reversing tumour immune suppression. An Fmoc group, an effective drug-interactive motif, is also introduced into the carrier to improve the drug loading capacity and formulation stability. We show that PEG_2k_-Fmoc-NLG alone is effective in enhancing T-cell immune responses and exhibits significant antitumour activity *in vivo*. More importantly, systemic delivery of paclitaxel (PTX) using the PEG_2k_-Fmoc-NLG nanocarrier leads to a significantly improved antitumour response in both breast cancer and melanoma mouse models.

## Abstract (web summary)

The use of immunostimulatory agents to enhance the efficacy of chemotherapy is a promising strategy in cancer therapy. Here, the authors report on a micellar nanoparticle that can effectively co-deliver chemo- and immunotherapeutics, resulting in an improved *in vivo* antitumour response.

## Graphical abstract

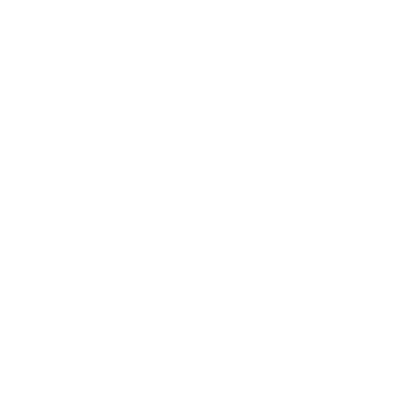

Chemotherapy remains a mainstay treatment for various types of cancers^1^,^2^. It is generally believed that chemotherapeutics work through cytostatic and/or cytotoxic effects^3^. Accumulating evidence suggests that chemotherapy-elicited immune responses also contribute significantly to the overall antitumour activity^4^,^5^,^6^,^7^. Chemotherapeutic agents can modify the propensity of malignant cells to elicit an immune response and/or directly exert immunostimulatory effects^8^. However, the effectiveness of chemotherapy-elicited immune response as well as other types of immunotherapies is limited by various negative feedback mechanisms that are upregulated during tumour development and/or cancer treatment^9^,^10^. Blockade of these negative regulatory pathways represents one of the most promising strategies to reactivate the immune response to cancer. Indeed, exciting preclinical and clinical anti-cancer results have been reported with the use of monoclonal antibodies against cytotoxic T-lymphocyte-associated protein 4 (CTLA-4) and programmed cell death protein 1 (PD-1), two important immune checkpoints^10^,^11^,^12^,^13^,^14^,^15^,^16^. Indoleamine 2,3-dioxygenase (IDO) is another important negative feedback protein involved in generating the immunosuppressive microenvironment that supports tumour cell growth^17^,^18^. IDO is overexpressed in some cancer cells and functions as an enzyme that catalyses the degradation of essential amino acid tryptophan (Trp) and accumulation of its metabolites^18^,^19^, resulting in cell cycle arrest and death of effector T cells, but increases in the number of regulatory T cells^20^.

Consistent with previous findings, we have indeed shown that the mRNA expression of IFN-γ was significantly increased in 4T1.2 tumour tissues following treatment with Taxol (Supplementary Fig. 1a). We have also found that Taxol treatment led to significant upregulation of IDO expression in tumour tissues (Supplementary Fig. 1b), suggesting that IDO was induced, likely by IFN-γ, to counterbalance antitumour immune responses. Thus, strategies that are targeted at IDO represent an attractive approach for the treatment of cancer, particularly in combination with chemotherapy^6^.

Several IDO inhibitors have been reported, among which NLG919 is a highly IDO-selective inhibitor with an EC50 of 75 nM. However, most IDO inhibitors including NLG919 are poorly water soluble, which presents a major challenge in their therapeutic delivery and preclinical evaluations^21^. In addition, co-delivery of IDO inhibitors and chemotherapeutic agents to tumours remains a challenge due to their different physical and pharmacokinetic profiles. To resolve these challenges, we develop a novel micellar nanocarrier that is based on PEG-derivatized NLG919 prodrug. An Fmoc group is also introduced into the conjugate based on our recent discovery of Fmoc as a ‘formulation chemophor' or a structural unit capable of interacting with many pharmaceutical agents^22^. We have recently shown that incorporation of an Fmoc motif into a micellar system can not only improve the drug loading capacity and formulation stability but also broaden its utility in formulating various therapeutic agents of diverse structures^23^,^24^. We hypothesize that PEG-Fmoc-NLG represents an immunostimulatory dual-functional nanocarrier that facilitates co-delivery with a chemotherapeutics and improves cancer immunochemotherapy. Indeed, we show that PEG_2k_-Fmoc-NLG alone is effective in enhancing T-cell immune responses and exhibits significant antitumour activity *in vivo*. More importantly, systemic delivery of PTX using the PEG_2k_-Fmoc-NLG nanocarrier leads to a significantly improved antitumour effect in both breast cancer and melanoma mouse models.

## Results

### Characterization of PEG_2k_-Fmoc-NLG

**Figure 1:**
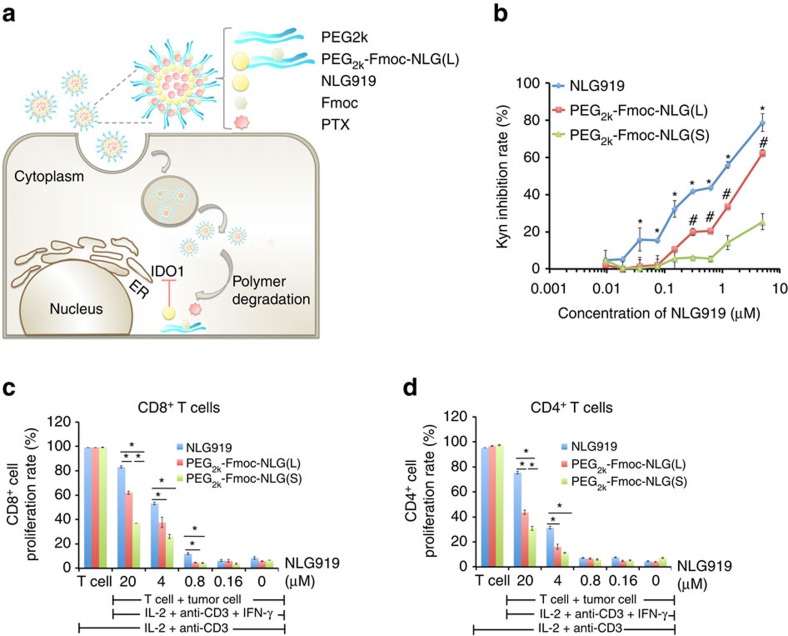
*In vitro* biological activities of PEG_2k_-Fmoc-NLG. (**a**) Schematic representation of self-assembled PTX/PEG_2k_-Fmoc-NLG mixed micelles. (**b**) PEG_2k_-Fmoc-NLG inhibited IDO enzyme activity *in vitro*. HeLa cells were treated with IFN-γ together with free NLG919 or PEG-NLG conjugate. Kynurenine in supernatants was measured 2 days later. Data represent means±s.e.m. *P<0.05 (versus PEG_2k_-Fmoc-NLG(L), *N*=3), ^#^*P*<0.05 (versus PEG_2k_-Fmoc-NLG(S), *N*=3). (**c**,**d**) IDO1 inhibition reversed T-cell suppression mediated by IDO-expressing mouse pancreatic cancer cells (Panc02). Panc02 cells and splenocytes were mixed and treated with IL-2, anti-CD3 antibody, IFN-γ together with NLG919 or PEG-NLG conjugate for 3 days. (**c**) CD4^+^ and (**d**) CD8^+^ T-cell proliferation was examined by FACS analysis. Representative data of three independent experiments are presented as means±s.e.m. *P<0.05.

PEG_2k_-Fmoc-NLG is an amphiphilic molecule that self-assembles into micelles in aqueous solutions. The self-assembly of PEG_2k_-Fmoc-NLG and the loading of hydrophobic drugs into the PEG_2k_-Fmoc-NLG micelles are illustrated in Fig. 1a. Supplementary Fig. 2 shows the synthesis scheme of two PEG_2k_-Fmoc-NLG conjugates, one with a relatively labile ester linkage (PEG_2k_-Fmoc-NLG(L)) and the other one with a relatively stable amide linkage (PEG_2k_-Fmoc-NLG(S)). The chemical structures of the two conjugates were confirmed by NMR and mass spectrometry (MS) (Supplementary Figs 3–6).

The inhibitory activity of PEG_2k_-Fmoc-NLG(L) and PEG_2k_-Fmoc-NLG(S) on IDO was evaluated by examining their potency in inhibiting the conversion of Trp to kynurenine (Kyn) in HeLa cells^21^,^25^. HeLa cells were treated with IFN-γ to induce IDO expression and the amounts of Trp and Kyn in culture medium were determined by a colorimetric assay. As shown in Fig. 1b, free NLG919 inhibited the IDO activity in a concentration-dependent manner with an EC50 of 0.95 μM. PEG_2k_-Fmoc-NLG(L) was less active (EC50 of 3.4 μM) in inhibiting IDO compared with free NLG919 while PEG_2k_-Fmoc-NLG(S) was least active (EC50>10 μM). Similar results were obtained when the Trp and Kyn concentrations were measured by high-performance liquid chromatography–mass spectrometry (HPLC–MS) (Supplementary Fig. 7). We then examined if inhibition of IDO by PEG_2k_-Fmoc-NLG(L) led to enhanced T-cell proliferation in an *in vitro* lymphocyte and Panc02 (a murine pancreatic cancer cell line) coculture experiment. As shown in Fig. 1c,d and Supplementary Fig. 8, coculture of IDO^+^ tumour cells with splenocytes isolated from BALB/c mice led to significant inhibition of T-cell proliferation. This inhibition was significantly attenuated when the mixed cells were treated with NLG919. PEG_2k_-Fmoc-NLG(L) was also active in reversing the inhibitory effect of tumour cells although slightly less potent than NLG919. PEG_2k_-Fmoc-NLG(S) is less active compared with PEG_2k_-Fmoc-NLG(L) (Fig. 1c,d; Supplementary Fig. 8).

**Figure 2:**
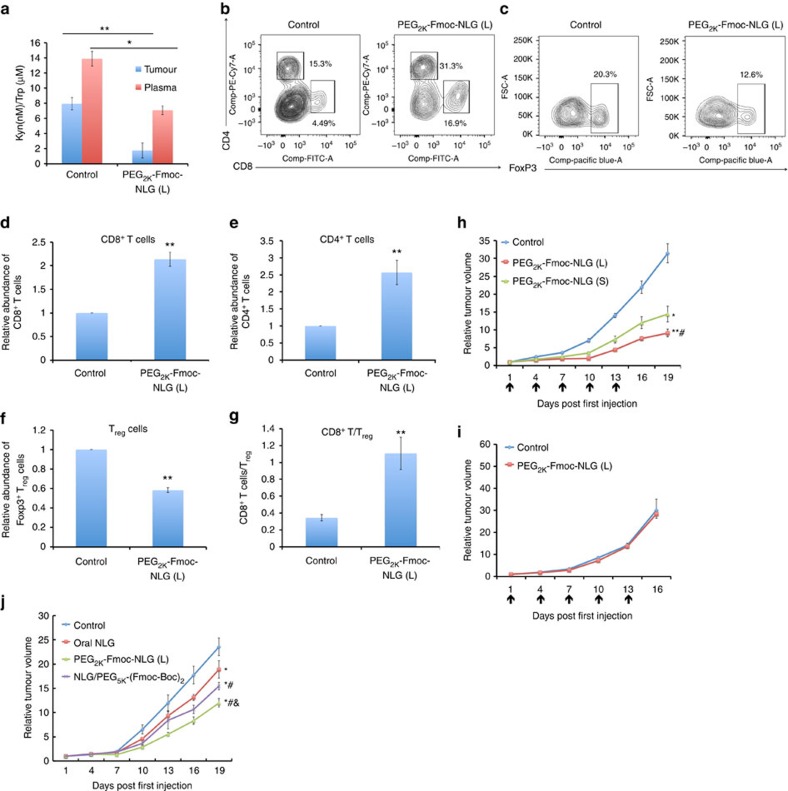
*In vivo* biological activities of PEG_2k_-Fmoc-NLG. (**a**) PEG_2k_-Fmoc-NLG(L) treatment decreased kynurenine concentrations in plasma and tumours. BALB/c mice bearing s.c. 4T1.2 tumours of ∼50 mm^3^ received PBS or PEG_2k_-Fmoc-NLG(L) i.v. once every 3 days for 5 times at a dose of 25 mg NLG919 per kg. Kynurenine/tryptophan ratios in plasma and tumours were determined by HPLC–MS one day following the last injection. Data are means±s.e.m. of 3 experiments. *P<0.05, **P<0.01. (**b**–**g**) IDO1 inhibition by PEG_2k_-Fmoc-NLG(L) increased CD4^+^ and CD8^+^ T cells, and decreased T_reg_ cells in tumours. Tumour-bearing mice were treated as described above. (**b**) Gating of CD8^+^ and CD4^+^ T cells (marked with black boxes) as a percentage of CD45^+^ lymphocytes. (**c**) Gating of T_reg_ (CD4^+^FoxP3^+^) cells (marked with black boxes) as a percentage of CD4^+^ lymphocytes. (**d**,**e**) Relative number of intratumoural CD8^+^ (**d**) and CD4^+^ (**e**) T cells following different treatments. (**f**,**g**) Relative number of T_reg_ cells (**f**) and CD8^+^ T cells/T_reg_ (**g**) in tumour tissues. Data represent means±s.e.m. (**P<0.01, *N*=5). (**h**) PEG_2k_-Fmoc-NLG maintained the tumour inhibitory effect. Mice bearing tumours of ∼50 mm^3^ received different treatments as indicated (black arrows). *P<0.05; **P<0.01 (versus control, *N*=5), ^#^*P*<0.05 (versus PEG_2k_-Fmoc-NLG(S), *N*=5). (**i**) Lymphocyte activities were required for the *in vivo* activity of PEG_2k_-Fmoc-NLG(L) micelles. Female BALB/c-nu/nu mice bearing 4T1.2 tumour of ∼50 mm^3^ were similarly treated as described above. (**j**) Enhanced *in vivo* antitumour activity of PEG_2k_-Fmoc-NLG(L) compared with oral delivery of NLG (^#^*P*<0.05, *N*=5) or NLG formulated in PEG_5k_-(Fmoc-Boc)_2_ micelles (^&^*P*<0.05, *N*=5). *P<0.05 (versus control, *N*=5). Data represent means±s.e.m.

The *in vivo* biological activity of PEG_2k_-Fmoc-NLG(L) was evaluated in an aggressive murine breast cancer model, 4T1.2. As detailed later, PEG_2k_-Fmoc-NLG(L) self-assembled to form nano-sized micelles (∼90 nm) in aqueous solutions, which shall enable effective and selective delivery to tumours via enhanced permeation and retention effect^26^. As shown in Fig. 2a, the ratios of Kyn (nM)/Trp (μM) in both blood and tumours were significantly reduced following the treatment of PEG_2k_-Fmoc-NLG(L) while a more dramatic reduction was observed in the tumour tissues, consistent with the intended preferential delivery of IDO inhibitors to the tumour tissues. Figure 2b–g shows multi-colour flow cytometric analysis of tumour-infiltrating lymphocytes in 4T1.2 tumour-bearing mice with or without treatment of PEG_2k_-Fmoc-NLG(L). It is clear that more CD4^+^ and CD8^+^ T cells were found in the tumours that received the treatment of PEG_2k_-Fmoc-NLG(L). In addition, the number of regulatory T cells (T_regs_) was significantly reduced in the tumours treated with PEG_2k_-Fmoc-NLG(L).

Figure 2h shows the *in vivo* antitumour activity of PEG_2k_-Fmoc-NLG(L) and PEG_2k_-Fmoc-NLG(S) in 4T1.2 tumour model. The tumour growth curves were presented as the relative tumour volumes. Data of actual tumour sizes for *in vivo* therapeutic study of carrier alone and other subsequent treatments were shown in Supplementary Figs 9 and 10. Significant antitumour responses were observed for both prodrugs (Fig. 2h; Supplementary Fig. 9a). It is also apparent that PEG_2k_-Fmoc-NLG(L) was more effective than PEG_2k_-Fmoc-NLG(S) in inhibiting the tumour growth (Fig. 2h; Supplementary Fig. 9a). We also showed that PEG_2k_-Fmoc-NLG(L) was essentially not active in inhibiting the growth of 4T1.2 tumour in the immunocompromised nude mice that lack T and B cells (Fig. 2i; Supplementary Fig. 9b), suggesting that the antitumour response was mediated via an enhanced T-cell immune response. The above data clearly demonstrated that PEG-derivatized NLG919 prodrug well retained the pharmacological activity of NLG919 and that the cleavability of NLG919 from the conjugate affected its activity.

We have further shown that i.v. PEG_2k_-Fmoc-NLG(L) was more effective than NLG919 delivered orally (Fig. 2j; Supplementary Fig. 9c). In addition, i.v. PEG_2k_-Fmoc-NLG(L) was more active than an i.v. formulation of NLG919 that was loaded into PEG_5k_-(Fmoc-Boc)_2_ micelles (Fig. 2j; Supplementary Fig. 9c).

### *In vitro* characterization of drug-loaded PEG_2k_-Fmoc-NLG micelles

**Figure 3:**
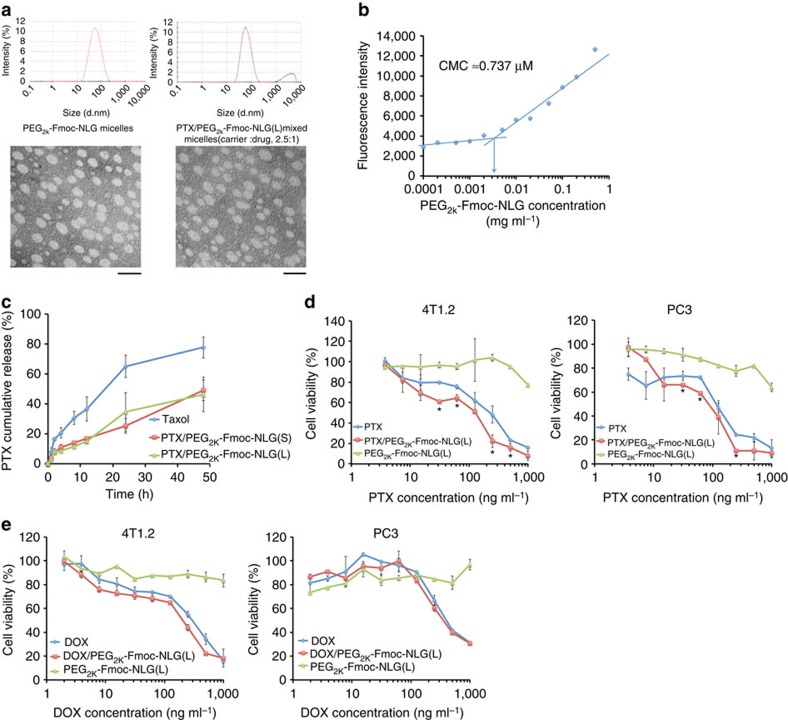
*In vitro* biophysical and biological characterizations of micelles. (**a**) Size distribution and morphology of drug-free and PTX-loaded PEG_2k_-Fmoc-NLG(L) micelles (carrier: drug, 2.5:1, m/m) were examined by dynamic light scattering and TEM, respectively. Drug concentration in micelles was kept at 1 mg ml^−1^. Blank micelle concentration was 20 mg ml^−1^. Scale bar, 100 nm. (**b**) Measurement of CMC of PEG_2k_-Fmoc-NLG(L) micelles. (**c**) PTX release kinetics of PTX/PEG_2k_-Fmoc-NLG(L) examined via a dialysis method. PTX concentrations were kept at 1 mg ml^−1^ in PTX/PEG_2k_-Fmoc-NLG(S), PTX/PEG_2k_-Fmoc-NLG(L) and Taxol. PTX concentrations were analysed at 0, 1, 2, 4, 8, 24 and 48 h by HPLC. (**d**) Cytotoxicity of PEG_2k_-Fmoc-NLG(L) alone, free PTX, and micellar PTX against a mouse breast cancer cell line (4T1.2) and a human prostate cancer cell line (PC3). Cells were treated for 72 h and cytotoxicity was determined by MTT assay. *P<0.05 (PTX/PEG_2k_-Fmoc-NLG(L) versus PTX), *N*=3. (**e**) Cytotoxicity of PEG_2k_-Fmoc-NLG(L) alone, free DOX, and micellar DOX against a mouse breast cancer cell line (4T1.2) and a human prostate cancer cell line (PC3). Data represent means±s.e.m.

PEG_2k_-Fmoc-NLG(L) readily formed small-sized (∼90 nm) micelles in aqueous solutions as confirmed by dynamic light scattering and transmission electron microscopy (TEM) imaging (Fig. 3a). Loading of PTX into PEG_2k_-Fmoc-NLG(L) micelles resulted in minimal changes in the sizes of the particles and their morphology (Fig. 3a). Similar results were obtained for PEG_2k_-Fmoc-NLG(S) micelles (data not shown). Figure 3b shows that the critical micelle concentration (CMC) of PEG_2k_-Fmoc-NLG(L) was 0.737 μM. The relatively low CMC may render the micelles stable upon dilution in the blood, which is important for systemic delivery to tumours. Table 1 shows the drug loading capacity of PEG_2k_-Fmoc-NLG(L) for several commonly used chemotherapeutic agents including PTX, docetaxel, doxorubicin (DOX), gefitinib, imatinib and curcumin. The effectiveness of PEG_2k_-Fmoc-NLG(L) in formulating various anticancer agents of diverse structures is likely attributed to the strong carrier/drug interactions including hydrophobic/hydrophobic interaction, *π*–*π* stacking and hydrogen bonding.

Figure 3c shows the kinetics of PTX release from PTX/PEG_2k_-Fmoc-NLG in comparison with Taxol. Taxol showed a relatively fast release of PTX with greater than 60% of PTX being released within 24 h. Close to 80% of PTX was released from Taxol after 48 h. In contrast, the kinetics of PTX release was significantly slower for either PTX/PEG_2k_-Fmoc-NLG(L) or PTX/PEG_2k_-Fmoc-NLG(S) formulation. Only 20–30% of PTX was released within 24 h and more than 50% of the PTX remained associated with the micelles after 48 h. Nonetheless, release of PTX from either PTX/PEG_2k_-Fmoc-NLG(L) or PTX/PEG_2k_-Fmoc-NLG(S) was significantly faster than the cleavage and release of NLG from either carrier; free NLG was essentially undetectable during the entire release study (data not shown).

Figure 3d shows the cytotoxicity of PTX-loaded PEG_2k_-Fmoc-NLG(L) in 4T1.2 cells. PEG_2k_-Fmoc-NLG(L) alone was not effective in inhibiting the tumour cell growth at the test concentrations. Free PTX inhibited the tumour cell growth in a concentration-dependent manner. PTX-loaded PEG_2k_-Fmoc-NLG(L) micelles were more effective (*P*<0.05) than free PTX at several concentrations tested (Fig. 3d). Similar results were found in the PC3 human prostate cancer cell line (Fig. 3d). We also observed enhanced cytotoxicity (*P*=0.053) for DOX following incorporation into PEG_2k_-Fmoc-NLG(L) micelles (Fig. 3e). The IC50s of free drugs (PTX or DOX) and drug-loaded micelles are shown in Table 2.

### Pharmacokinetics and biodistribution

**Figure 4:**
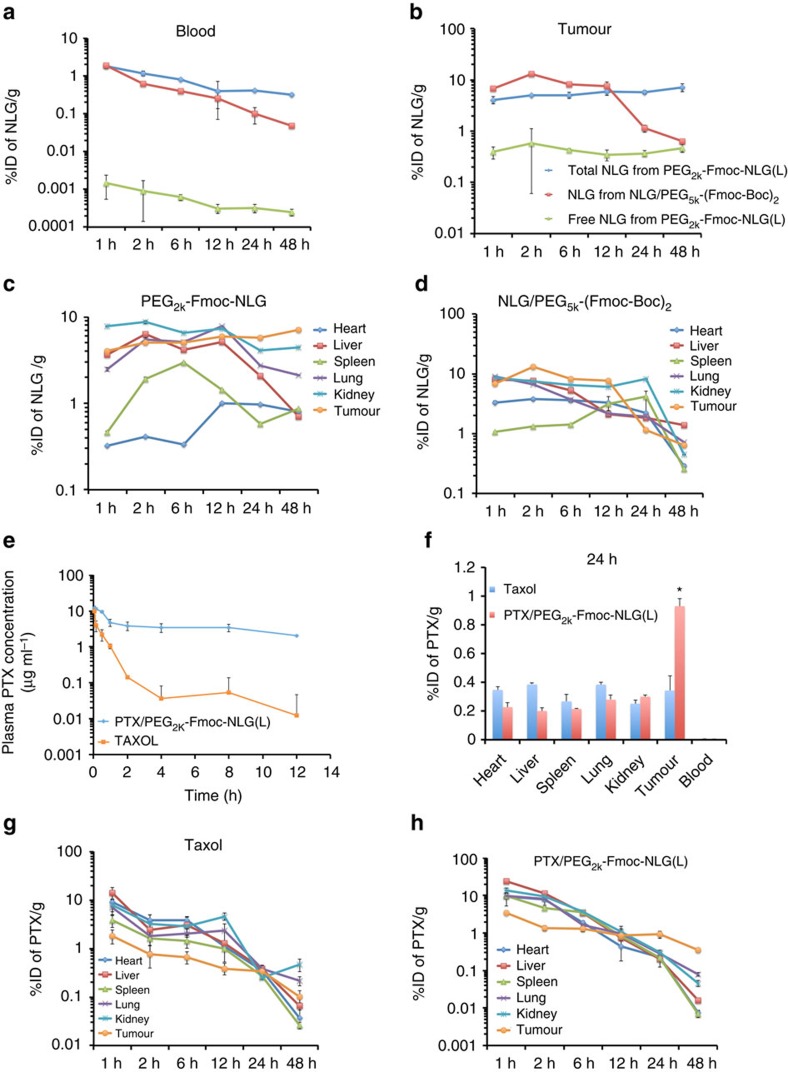
Pharmacokinetics and biodistribution of drug-free and PTX-loaded micelles. (**a**,**b**) The kinetics of NLG in blood (**a**) and tumour (**b**) in 4T1.2 tumour-bearing mice following i.v. administration of PEG_2k_-Fmoc-NLG(L) in comparison to NLG-loaded PEG_5k_-(Fmoc-Boc)_2_ micelles (25 mg NLG per kg). (**c**,**d**) Tissue distribution of NLG in 4T1.2 tumour-bearing BALB/c mice following i.v. administration of PEG_2k_-Fmoc-NLG(L) (**c**) or NLG-loaded PEG_5k_-(Fmoc-Boc)_2_ micelles (**d**) at a NLG dose of 25 mg kg^−1^. (**e**) Blood kinetics of PTX in BALB/c mice following i.v. administration of Taxol or PTX/PEG_2k_-Fmoc-NLG(L) mixed micelles at a dose of 10 mg PTX per kg. (**f**) Tissue distribution of PTX in 4T1.2 tumour-bearing BALB/c mice 24 h following i.v. administration of Taxol or PTX/PEG_2k_-Fmoc-NLG(L) mixed micelles at a PTX dose of 10 mg kg^−1^. *P<0.05 (*N*=5). (**g**,**h**) Tissue distributions of PTX at various time points following i.v. administration of Taxol (**g**) or PTX/PEG_2k_-Fmoc-NLG(L) mixed micelles (**h**) (10 mg PTX per kg). All data represent means±s.e.m.

Figure 4a shows the kinetics of PEG-Fmoc-NLG in the blood in comparison to NLG loaded into PEG_5k_-(Fmoc-Boc)_2_ micelles. The concentrations of total NLG (intact PEG_2k_-Fmoc-NLG plus released free NLG) in the blood were significantly higher than the blood concentrations of NLG delivered by PEG_5k_-(Fmoc-Boc)_2_ micelles at most time points examined. It is also apparent that very little free NLG was detected in the blood in the group treated with PEG_2k_-Fmoc-NLG, suggesting the excellent stability of the conjugate in the blood.

Figure 4b shows the amounts of total NLG in the tumours at different time points following i.v. administration of either PEG_2k_-Fmoc-NLG or NLG-loaded PEG_5k_-(Fmoc-Boc)_2_ micelles. The NLG concentrations in the tumours in NLG/PEG_5k_-(Fmoc-Boc)_2_ group reached the peak levels at 2 h and then quickly declined over time. In contrast, high concentrations of NLG (largely intact conjugate) were found in the tumours over the entire 48 h in the mice treated with PEG_2k_-Fmoc-NLG. It is also apparent that a relatively constant concentration of free NLG was detected in the tumours in this group, albeit at a low level, suggesting that NLG was slowly but continuously released from the conjugate over a prolonged period of time. Figure 4c,d shows the total amounts of NLG in tumours and other major organs/tissues at various times following i.v. administration of either PEG_2k_-Fmoc-NLG or NLG/PEG_5k_-(Fmoc-Boc)_2_ mixed micelles.

Figure 4e shows the blood PTX kinetics in BALB/c mice as a function of time following i.v. bolus administration of PTX-loaded PEG_2k_-Fmoc-NLG(L) and Taxol. It is apparent that PTX/PEG_2k_-Fmoc-NLG(L) remained in the circulation for a significantly longer time compared with Taxol. The pharmacokinetic parameters are outlined in Table 3. Incorporation of PTX into PEG_2k_-Fmoc-NLG(L) micelles resulted in significantly greater *t*_1/2_, area under curve (AUC), and *C*_max_ over Taxol. Meanwhile, volume of distribution (Vd) and clearance (CL) for PTX/PEG_2k_-Fmoc-NLG(L) were significantly lower than those for Taxol.

Figure 4f shows the biodistribution of PTX in 4T1.2 tumour-bearing mice 24 h following i.v. administration of PTX-loaded PEG_2k_-Fmoc-NLG(L) micelles or Taxol. Significantly greater amounts of PTX were found in tumour tissues for PTX-loaded PEG_2k_-Fmoc-NLG(L) micelles in comparison with Taxol. In contrast, PTX-loaded PEG_2k_-Fmoc-NLG(L) micelles showed significantly reduced accumulation than Taxol in liver, spleen and other organs/tissues. These data strongly suggest that PTX-loaded PEG_2k_-Fmoc-NLG(L) micelles are stable in the blood and are highly effective in selective delivery to the tumour tissues. Figure 4g,h shows the amounts of PTX in tumours and other major organs/tissues at various times following i.v. administration of either PTX-loaded PEG_2k_-Fmoc-NLG(L) micelles or Taxol.

### *In vivo* antitumour activity of PTX-loaded micelles

**Figure 5:**
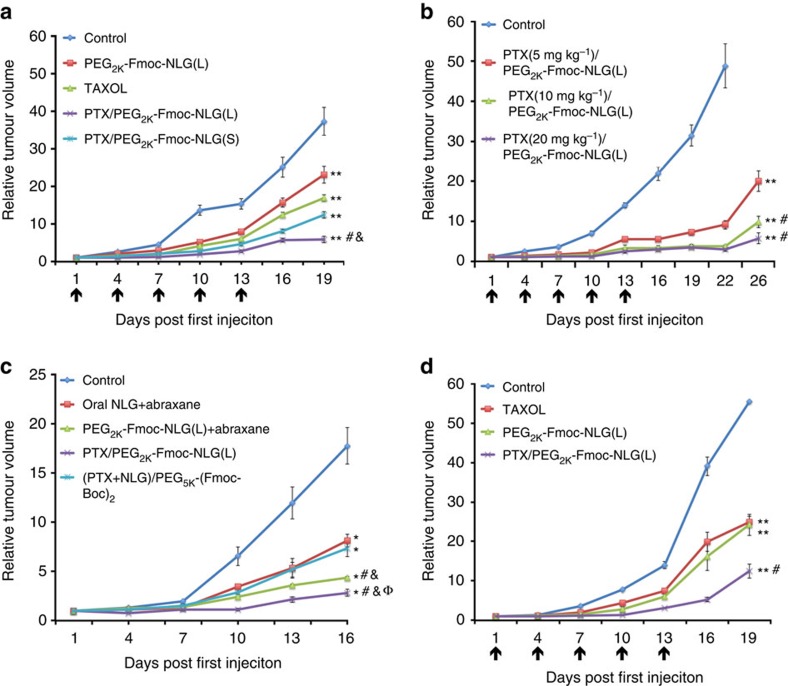
*In vivo* antitumour activity of PTX-loaded PEG_2k_-Fmoc-NLG micelles. (**a**) *In vivo* antitumour activity of various PTX formulations in 4T1.2 tumour model. PTX dose was 10 mg kg^−1^. Tumour sizes were plotted as relative tumour volumes. **P<0.01 (all treatment groups versus control group), ^#^*P*<0.05 (PTX/PEG_2k_-Fmoc-NLG(L) versus Taxol ), ^&^*P*<0.05 (PTX/PEG_2k_-Fmoc-NLG(L) versus PTX/PEG_2k_-Fmoc-NLG(S)). *N*=5. (**b**) Dose-escalation study on the antitumour activity of PTX-loaded PEG_2k_-Fmoc-NLG(L) micelles. PTX dose was 5, 10 and 20 mg kg^−1^, respectively. **P<0.01 (all treatment groups versus control), ^#^*P*<0.05 (10 mg, 20 mg PTX per kg versus 5 mg PTX per kg). *N*=5. (**c**) Antitumour activity of PTX/PEG_2k_-Fmoc-NLG(L) in a 4T1.2 tumour model in comparison to a combination of oral NLG with i.v. Abraxane, PEG_2k_-Fmoc-NLG(L) plus Abraxane or PEG_5k_-(Fmoc-Boc)_2_ micelles co-loaded with PTX and NLG. *P<0.01 (all treatment groups versus control), ^#^*P*<0.05 (PTX/PEG_2k_-Fmoc-NLG(L) or PEG_2k_-Fmoc-NLG(L)+Abraxane versus oral NLG+Abraxane), ^&^*P*<0.05 (PTX/PEG_2k_-Fmoc-NLG(L) or PEG_2k_-Fmoc-NLG(L)+Abraxane versus (PTX+NLG)/PEG_5k_-(Fmoc-Boc)_2_), ^Φ^*P*<0.05 (PTX/PEG_2k_-Fmoc-NLG(L) versus PEG_2k_-Fmoc-NLG(L)+Abraxane), *N*=5. (**d**) Antitumour activity of PTX/PEG_2k_-Fmoc-NLG(L) in a murine melanoma (B16) model. PTX dose was 10 mg kg^−1^. **P<0.01 (all treatment groups versus control), ^#^*P*<0.05 (PTX/PEG_2k_-Fmoc-NLG(L) versus Taxol), N=5. All data represent means±s.e.m.

Figure 5a and Supplementary Fig. 10a show the *in vivo* antitumour activity of PEG_2k_-Fmoc-NLG(L), Taxol, PTX/PEG_2k_-Fmoc-NLG(S) and PTX/PEG_2k_-Fmoc-NLG(L) at a PTX dosage of 10 mg kg^−1^. Taxol showed a modest effect in inhibiting the growth of 4T1.2 tumour, which was comparable to that of PEG_2k_-Fmoc-NLG(L) alone. However, both PTX/PEG_2k_-Fmoc-NLG(S) and PTX/PEG_2k_-Fmoc-NLG(L) were more effective than Taxol or PEG_2k_-Fmoc-NLG(L) in inhibiting the tumour growth. It is also apparent that PTX/PEG_2k_-Fmoc-NLG(L) was more effective than PTX/PEG_2k_-Fmoc-NLG(S), suggesting a role of released NLG919 in the overall antitumour activity of PTX/PEG_2k_-Fmoc-NLG(L). The antitumour activity of the three PTX formulations follows the order of PTX/PEG_2k_-Fmoc-NLG(L)>PTX/PEG_2k_-Fmoc-NLG(S)>Taxol≈PEG_2k_-Fmoc-NLG(L).

The effective inhibition of tumour growth by PTX/PEG_2k_-Fmoc-NLG(L) was associated with a significant survival benefit (Supplementary Fig. 11). The median survival time of mice in PTX/PEG_2k_-Fmoc-NLG(L) group is significantly longer compared with the mice treated with Taxol (*P*<0.01) or PEG_2k_-Fmoc-NLG(L) (*P*<0.01).

Figure 5b and Supplementary Fig. 10b show the antitumour activity of PTX/PEG_2k_-Fmoc-NLG(L) at various doses of PTX. Tumour growth was well controlled at all dose groups at early time points. After the last treatment at day 13, the tumour growth was almost stalled until day 22 for the groups of 10 and 20 mg PTX per kg. After that, there was a rebound in tumour growth, particularly in the low-dose group.

Figure 5c and Supplementary Fig. 10c show that PTX/PEG_2k_-Fmoc-NLG(L) was also more effective than a combination therapy that involves oral delivery of NLG together with i.v. administration of Abraxane. In addition, PTX/PEG_2k_-Fmoc-NLG(L) was more active than a combination of i.v. Abraxane with i.v. PEG_2k_-Fmoc-NLG(L). Furthermore, PTX/PEG_2k_-Fmoc-NLG(L) was more active than an i.v. formulation of PEG_5k_-(Fmoc-Boc)_2_ that was co-loaded with PTX and NLG. Improved antitumour activity of PTX/PEG_2k_-Fmoc-NLG(L) was also demonstrated in an aggressive B16 murine melanoma model (Fig. 5d; Supplementary Fig. 10d).

All of the treatments were well tolerated by the mice and there were no abnormal physical signs in all treated mice. In addition, there were no obvious differences among all of the groups in body weights in all of the different therapy studies (Supplementary Fig. 12).

**Figure 6:**
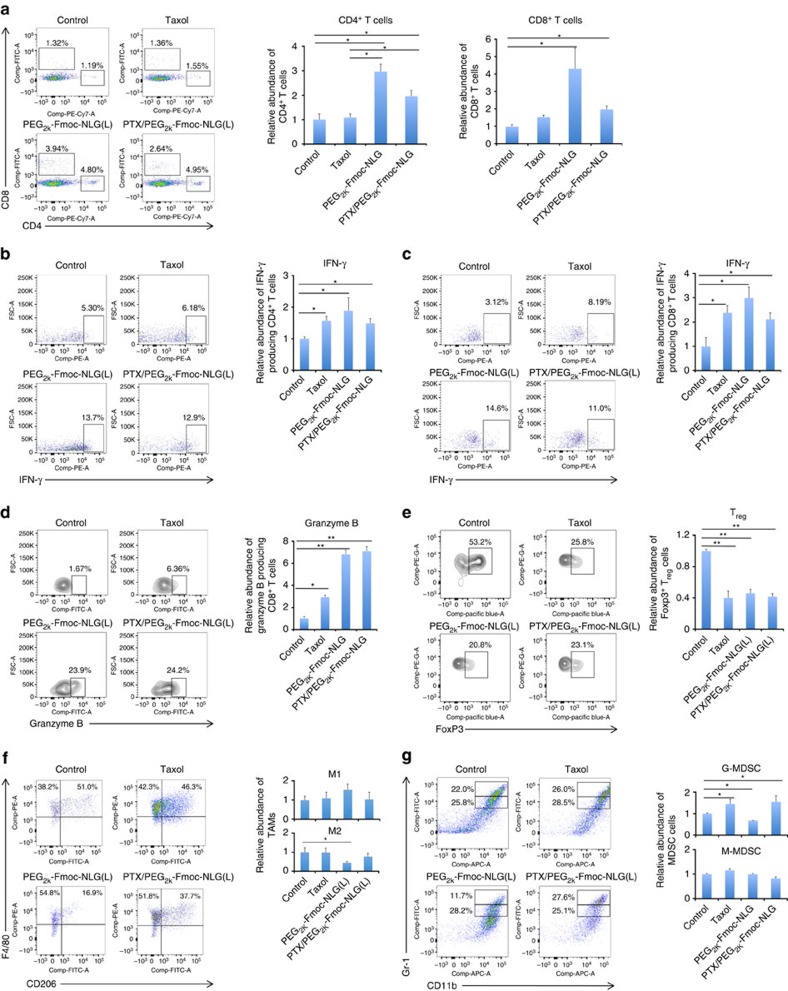
Flow cytometry analysis of immune cell subsets in tumour tissues. (**a**–**d**) T-cell infiltration in mouse tumours treated with Taxol, PEG_2k_-Fmoc-NLG(L) or PTX/PEG_2k_-Fmoc-NLG(L) at a PTX dosage of 10 mg kg^−1^. The relative abundance of CD4^+^, CD8^+^ (**a**), IFN-γ positive intratumoural CD4^+^ T cells (**b**), IFN-γ positive intratumoural CD8^+^ T cells (**c**), and granzyme B-positive CD8^+^ T cells (**d**) in tumour tissues were detected by flow cytometry. (**e**) Flow cytometry gating and histogram analysis of FoxP3^+^ T regulatory cells in mouse tumours. (**f**) Tumour-associated macrophages (TAMs) in mouse tumours. The percentages of TAM populations with specific macrophage markers (M1-type (CD11b^+^/F4/80^+^/CD206^−^) and M2-type (CD11b^+^/F4/80^+^/CD206^+^)) in tumour tissues were detected by flow cytometry. (**g**) Flow cytometry gating and histograms analysis of CD11b^+^/Gr-1^+^ MDSC cells in mouse tumours. Double positive cells contain two populations, including Gr-1^high^CD11b^+^ granulocytic (G-MDSC) and Gr-1^int^CD11b^+^ monocytic (M-MDSC) MDSC subsets. The bars represent means±s.e.m. (*P<0.05, **P<0.01, *N*=3).

To delineate a role of immune response in PTX/PEG_2k_-Fmoc-NLG(L)-mediated antitumour activity, the immune cell populations in the tumour tissues with various treatments were analysed by flow cytometry one day following five times of treatments. Figure 6a shows infiltration of more CD4^+^ T cells in the tumours treated with PTX/PEG_2k_-Fmoc-NLG(L) compared with control or Taxol groups (*P*<0.05). There were also more CD8^+^ T cells in the tumours treated with PTX/PEG_2k_-Fmoc-NLG(L) compared with control group. It was also noted that the numbers of both CD4^+^ and CD8^+^ T cells in Taxol-treated tumours were lower than those in the tumours treated with carrier alone (Fig. 6a). Delivery of PTX via PEG_2k_-Fmoc-NLG(L) was associated with a similar reduction in the numbers of CD4^+^ and CD8^+^ T cells (Fig. 6a).

Figure 6b,c shows that the numbers of IFN-γ-positive CD4^+^ or CD8^+^ T cells were significantly increased in the tumours treated with Taxol, PEG_2k_-Fmoc-NLG(L) or PTX/PEG_2k_-Fmoc-NLG(L). The magnitude of increase was similar among all of the treatment groups.

The numbers of granzyme B-positive CD8^+^ T cells were also significantly increased in all of the treatment groups (Fig. 6d). However, there were significantly more granzyme B-positive CD8^+^ T cells in the tumours treated with PEG_2k_-Fmoc-NLG(L) or PTX/PEG_2k_-Fmoc-NLG(L) compared with Taxol-treated tumours (Fig. 6d). There were no differences between PEG_2k_-Fmoc-NLG(L) and PTX/PEG_2k_-Fmoc-NLG(L) groups in the numbers of granzyme B-positive CD8^+^ T cells (Fig. 6d).

T_reg_ cells were significantly decreased in all treatment groups compared with control group (*P*<0.01) and there were no significant differences among these treatment groups (*P*>0.05) (Fig. 6e).

Figure 6f shows that the numbers of M2 (CD11b^+^F4/80^+^CD206^+^) tumour-associated macrophages were significantly reduced in the tumours treated with PEG_2k_-Fmoc-NLG(L). Meanwhile, the numbers of M1 (CD11b^+^F4/80^+^CD206^−^) tumour-associated macrophages were slightly increased. There were no significant changes in the numbers of either M1 or M2 macrophages in the tumours treated with Taxol or PTX/PEG_2k_-Fmoc-NLG(L). Similar results were obtained in an experiment in which CD11b^+^F4/80^+^CD86^+^ were used to define M1 macrophages (Supplementary Fig. 13).

Figure 6g shows that the numbers of granulocytic myeloid-derived suppressor cells (G-MDSC) were significantly decreased in the tumours treated with PEG_2k_-Fmoc-NLG(L) alone. This is consistent with the previous reports that inhibition of IDO leads to decreased MDSC in the tumours^27^,^28^. Surprisingly, G-MDSC were significantly increased in the tumours treated with either PTX/PEG_2k_-Fmoc-NLG(L) or Taxol. There were no significant differences among all of the groups in the numbers of monocytic MDSC (M-MDSC) in the tumours (Fig. 6g).

The immune cell populations in the tumour tissues were also examined at an earlier time point (one day following the first injection) and similar results were obtained (Supplementary Fig. 14).

Supplementary Fig. 15 shows the histology of tumours at the time of flow cytometry analysis (one day following 5 injections). Tumours from the mice treated with PTX/PEG_2k_-Fmoc-NLG(L) exhibited significant necrosis/apoptosis of tumour cells. Tumours treated with Taxol or PEG_2k_-Fmoc-NLG also showed moderate tumour cell damage.

Overall, the above data suggest that the microenvironment in the tumours treated with PTX/PEG_2k_-Fmoc-NLG(L) was more immune-active than that in Taxol-treated tumours. This is consistent with the data that the *in vivo* IDO activity was more effectively inhibited in mice treated with PTX/PEG_2k_-Fmoc-NLG(L) compared with Taxol-treated mice (Supplementary Fig. 16).

## Discussion

We have developed a rational and effective immunochemotherapy approach that is based on PEG-NLG919-mediated co-delivery of PTX. Different from most drug carriers that are ‘inert', PEG-Fmoc-NLG is a prodrug that exhibits immunostimulatory activity. Despite its reduced EC50 compared with free NLG with respect to the potency in inhibiting IDO in cultured cells, PEG-Fmoc-NLG was significantly more effective than NLG that was formulated in a similar ‘inert' nanocarrier without a NLG motif (PEG_5k_-(Fmoc-Boc)_2_) (Fig. 2j). In addition, i.v. PEG-Fmoc-NLG was more active than NLG delivered orally (Fig. 2j). This is likely due to the effective delivery of PEG-Fmoc-NLG to the tumours (Fig. 4b,c). The slow release of NLG from PEG-Fmoc-NLG in tumour tissues (Fig. 4b) may also play a role.

A major advantage of our approach is simultaneous delivery to the tumours of two agents of different mechanisms of action. In addition, this system could provide a programmable release of various drug components via both chemical conjugation and physical encapsulation. PTX and NLG showed different temporal release kinetics upon co-delivery to tumours. PTX has a much faster rate of release compared with that of NLG (Fig. 3c). PEG-Fmoc-NLG also has a longer retention time in the tumours (Fig. 4b), likely due to its macromolecule nature. Delivery of PTX via PEG-Fmoc-NLG was more effective in inhibiting the tumour growth than co-delivery of PTX and NLG via a similar ‘inert' nanocarrier without a NLG motif (Fig. 5c). In addition, PTX/PEG_2k_-Fmoc-NLG(L) was more effective than oral delivery of NLG together with i.v. administration of Abraxane (Fig. 5c). We hypothesize that the relatively rapid release of PTX will lead to the first round of antitumour response that will be further potentiated by the immune response that follows. The immune response could result from enhanced antigen presentation following PTX-mediated killing of tumour cells and/or direct effect of PTX on immune cells^8^. Meanwhile, the slow release of active NLG919 from the prodrug helps sustaining or enhancing the magnitude of immune responses by reversing IDO-mediated immune suppression. As a result, the combined therapy has produced a substantial inhibition of tumour growth. In fact, PTX/PEG_2k_-Fmoc-NLG(L) outperformed most reported PTX formulations including PTX formulated in our non-immunostimulatory dual-functional carriers^22^,^29^. It is possible that the carrier-mediated antitumour activity can be further improved via incorporation of a tumour microenvironment-responsive linkage^29^ to facilitate the NLG release. Another advantage of our strategy lies in the simplicity with respect to both the synthesis of dual-function carrier and the combination therapy protocol, which is expected to facilitate a rapid translation into clinic. In addition, our nanocarrier is versatile in formulating various anticancer agents of diverse structures (Table 1).

Immunological analysis indicates that, compared with those tumours from the no-treatment group, tumour tissues isolated from mice treated with PEG_2k_-Fmoc-NLG(L) are more immunoactive with more functional CD4^+^ and CD8^+^ T cells, decreased T_reg_ and MDSC, and increased M1/M2 ratios. In addition, we showed a more immunoactive microenvironment within tumours treated with either Taxol or PTX/PEG_2k_-Fmoc-NLG(L) compared with the no-treatment group in most of the parameters examined, indicating immunogenic function of these compounds^30^,^31^. However, tumour tissues from Taxol or PTX/PEG_2k_-Fmoc-NLG(L) groups had lower percentages of T cells including Treg, CD4^+^ and CD8^+^ T cells, but higher percentage of MDSC than the group treated with PEG_2k_-Fmoc-NLG(L) alone. The reduction in the percentage of total intratumoural T cells in the PTX/PEG_2k_-Fmoc-NLG(L) group relative to the PEG_2k_-Fmoc-NLG(L) group could be due to the cytotoxic effect of PTX treatment on these immune cells. Alternatively, it can also be attributed to the direct effect of PTX on tumour cells, leading to reduced production of inflammatory factors. Nonetheless, when compared with PEG_2k_-Fmoc-NLG(L) treatment, PTX/PEG_2k_-Fmoc-NLG(L) does not significantly affect the percentage of T cells that produce IFN-γ or Granzyme B, suggesting the antitumour effector function of intratumoural T cells is not affected by PTX. In addition, the cytotoxic effect of PTX might lead to reduced tumour burden, which can help enhance the overall efficacy of immunochemotherapy. Moreover, tumours treated with PTX/PEG_2k_-Fmoc-NLG(L) had more granzyme B-producing CD8^+^ T cells than the tumours treated with Taxol, suggesting IDO inhibition can still enhance antitumour T-cell immune responses in spite of repeated chemotherapy. Overall, our *in vivo* results show that the tumoricidal activity of PTX and the immune-enhancing function of NLG synergistically produced much more profound antitumour efficacy.

It should be noted that our strategy does not preclude the development of an oral NLG-based treatment. The purpose of our comparative study (Figs 2j and 5c) is to show the advantages of our strategy over other approaches at similar doses with respect to both the simplicity and potency as far as a combination therapy with a chemotherapeutic agent is concerned. Eventually, a therapeutic regimen that involves systemic immunochemotherapy followed by oral NLG-based sustained treatment can be developed to maximize the treatment outcome.

In summary, we have developed a simple and rational co-delivery approach that is effective in improving cancer immunochemotherapy. Although most of the works in this study are focused on PTX, it can be readily extended to immunochemotherapy with other anticancer agents such as DOX. Finally, such strategy can be employed in novel cancer therapy combining chemotherapy drugs and other immune modulating agents such as small molecule inhibitors of PD-1.

## Methods

### Reagents

Paclitaxel (PTX, >99%) was purchased from TSZ Chem (MA, USA). Docetaxel (DTX, >99%) was obtained from LC Laboratories (MA, USA). α-Fmoc-ɛ-Boc-lysine, N, N'-dicyclohexylcarbodiimide (DCC), trifluoroacetic acid (TFA) and triethylamine (TEA) were purchased from Acros Organic (NJ, USA). Monomethoxy PEG_2000_, 4-dimethylaminopyridine (DMAP), ninhydrin, and other unspecified chemicals were all purchased from Sigma Aldrich (MO, USA). Dulbecco's phosphate buffered saline (DPBS), Dulbecco's Modified Eagle's Medium (DMEM), fetal bovine serum (FBS), penicillin-streptomycin solution (100x) were all purchased from Invitrogen (NY, USA). All solvents used in this study were HPLC grade.

### Animals

Female BALB/c mice (4–6 weeks), female BALB/c nude mice (4–6 weeks) and C57BL/6 mice (4–6 weeks) were purchased from Charles River (Davis, CA). All animals were housed under pathogen-free conditions according to AAALAC (Association for Assessment and Accreditation of Laboratory Animal Care) guidelines. All animal-related experiments were performed in full compliance with institutional guidelines and approved by the Animal Use and Care Administrative Advisory Committee at the University of Pittsburgh.

### Cell culture

4T1.2 murine breast cancer cells, B16 murine melanoma cells, Panc02 murine pancreatic ductal adenocarcinoma cells, HeLa human cervical cancer cells, and PC3 human prostate cancer cells were maintained in Dulbecco's Modified Eagle's Medium (DMEM) supplemented with 10% fetal bovine serum (FBS) and 1% penicillin-streptomycin at 37 °C in a humidified environment with 5% CO_2_. All cell lines used in this work were obtained from ATCC (Manassas, VA).

### Synthesis of PEG_2k_-Fmoc-NLG conjugate

Both PEG_2k_-Fmoc-NLG(L) and PEG_2k_-Fmoc-NLG(S) conjugates were synthesized by coupling NLG919 to PEG_2k_ with either an ester or ether linkage. PEG_2k_-Fmoc-NLG(L) was synthesized as follow: 1 equiv. of monomethoxy PEG_2000_ was mixed with 3 equiv. of α-Fmoc-ɛ-Boc-lysine and DCC in dichloromethane (DCM) in the presence of DMAP for 2 days at room temperature (RT). Purified PEG_2K_-Fmoc-lysine-Boc was obtained by filtering the mixture and then precipitation with ice-cold ether/ethanol twice. The Boc group was removed by treatment with DCM/TFA (1:1, v/v) for 2 h at RT and the deprotected PEG_2K_-lysine(Fmoc)-NH_2_ was obtained by precipitation with ice-cold ether/ethanol. Finally, PEG_2k_-Fmoc-NLG(L) was synthesized by mixing PEG_2k_-lysine(Fmoc)-NH_2_ with excess amount of NLG919, DCC and small amount of DMAP in DCM at RT for 2 days. The mixture was filtered and the filtrate was precipitated by ice-cold ether/ethanol twice. The crude product was dissolved in water and filtered through a 450 nm filter, followed by lyophilization to yield the powder of purified PEG_2k_-Fmoc-NLG(L). To synthesize PEG_2k_-Fmoc-NLG(S), NLG919 was reacted with methyl 4-bromobutanoate to form ether bond under NaH condition. After column purification, the methyl ester was hydrolysed by NaOH and the obtained compound (3 equiv.) was conjugated with PEG_2k_-lys(Fmoc)-NH_2_ (1 equiv.) using DCC (3 equiv.) and DMAP (0.3 equiv.). The mixture was filtered and the clear filtrate was precipitated by ice-cold ether/ethanol twice. The crude product was dissolved in water, filtered, and lyophilized to obtain the purified PEG_2k_-Fmoc-NLG(S).

### Cell-based IDO assays

The IDO inhibitory effect of PEG_2k_-Fmoc-NLG was tested by an *in vitro* IDO assay^21^. Briefly, HeLa cells were seeded in a 96-well plate at a cell density of 5 × 10^3^ cells per well and allowed to grow overnight. Recombinant human IFN-γ was then added to each well with a final concentration of 50 ng ml^−1^. At the same time, various concentrations of PEG_2k_-Fmoc-NLG(L), PEG_2k_-Fmoc-NLG(S) or free NLG919 (NLG919 concentrations: 50 nM–20 μM) were added to the cells. After 48 h of incubation, 150 μl of the supernatants per well was transferred to a new 96-well plate. Seventy-five microliter of 30% trichloroacetic acid was added into each well and the mixture was incubated at 50 °C for 30 min to hydrolyse N-formylkynurenine to kynurenine. For colorimetric assay, supernatants were transferred to a new 96-well plate, mixed with equal volume of Ehrlich reagent (2% p-dimethylamino-benzaldehyde w/v in glacial acetic acid), and incubated for 10 min at RT. Reaction product was measured at 490 nm by a plate reader. For HPLC–MS/MS detection (Wastes Alliance 2695 Separation Module combined with Waters Micromass Quattro Micro TM API MS detector), the plate was centrifuged for 10 min at 2,500 r.p.m. and 100 μl of the supernatants per well was collected for tryptophan and kynurenine assay.

### T-cell proliferation study

A lymphocyte-Panc02 cell co-culture study was conducted to examine whether PEG_2k_-Fmoc-NLG can reverse IDO1-mediated inhibition of T-cell proliferation^21^,^25^. Murine Panc02 cells were stimulated by IFN-γ (50 ng ml^−1^) to induce IDO expression and then irradiated (6,000 rad) before coculture. Splenocyte suspensions were generated from BALB/c mice by passage through the nylon wool columns after lysing of red blood cells. IFN-γ-stimulated Panc02 cells (1 × 10^5^ cells per well) were mixed with splenocytes (5 × 10^5^ cells per well, pre-stained with 5-(and 6)-carboxyfluorescein diacetate (CFSE)) in a 96-well plate. Various concentrations of NLG919, PEG_2k_-Fmoc-NLG(L) or PEG_2k_-Fmoc-NLG(S) were added to the cells. To measure the T-cell proliferation, 100 ng ml^−1^ anti-CD3 and 10 ng ml^−1^ mouse recombinant IL-2 were added to the cocultures. The proliferation of CD8^+^ and CD4^+^T cells was measured by fluorescence-activated cell sorting (FACS) analysis after 3 days of coculture.

### Measurements of Trp and Kyn in plasma and tumour tissues

The kynurenine to tryptophan ratios in plasma or tumours in 4T1.2 tumour-bearing mice following different treatments were examined by HPLC–MS/MS as an indication of IDO enzyme activity^32^. BALB/c mice bearing 4T1.2 tumours of ∼50 mm^3^ were treated with DPBS, TAXOL (10 mg PTX per kg), PEG_2k_-Fmoc-NLG(L), or PTX/PEG_2k_-Fmoc-NLG(L) (10 mg PTX per kg) via tail vein once every 3 days for 5 times. One day after the last treatment, the plasma and tumour samples were harvested. Plasma samples were mixed with methanol (plasma: methanol, 1:2.5, v/v) and centrifuged at 14,500 r.p.m. for 15 min. Supernatants were collected for HPLC–MS quantification of kynurenine and tryptophan.

Tumour samples were homogenized in water and the homogenates were mixed with acetonitrile (1:1, v/v), centrifuged and supernatants were transferred to clean tubes. Equal volumes of methanol were added to precipitate proteins and supernatants were collected following centrifugation for HPLC–MS/MS measurement.

### Quantification of tumour-infiltrating lymphocytes

BALB/c mice bearing 4T1.2 tumours of ∼50 mm^3^ received various treatments via tail vein injection once every 3 days for 5 times. Tumours and spleen were harvested one day following the last treatment. Single cell suspensions were prepared and costained for CD4, CD8, IFN-γ, Granzyme B, FoxP3, myloid-derived suppressor cell (CD11b and Gr-1) and macrophage (F4/80 and CD206) for FACS analysis.

### *In vivo* therapeutic study of PEG_2k_-Fmoc-NLG micelles

To investigate whether IDO1 inhibition by PEG_2k_-Fmoc-NLG micelles can suppress tumour growth, female BALB/c mice of 4–6 weeks old were s.c. inoculated with 4T1.2 tumour cells (2 × 10^5^ cells per mouse)^20^,^33^. Mice were randomly grouped (*N*=5) when the tumour volume reached ∼50 mm^3^ and treated with PEG_2k_-Fmoc-NLG(L), PEG_2k_-Fmoc-NLG919(S), or NLG formulated in PEG5k-(Fmoc-Boc)_2_ micelles (25 mg NLG919 per kg) once every 3 days for 5 times via tail vein injection. A separate group was treated with NLG919 orally once daily for 15 days. Tumour sizes were measured twice weekly in two dimensions using a caliper, and the tumour volumes were calculated with the formula: V=(A × B^2^)/2 (A and B are the long and short diameters of the tumour). Relative tumour volume was calculated to compare different treatment groups. The maximum allowable tumour size is 20 mm in diameter in the animal protocol. Mice were sacrificed when tumour volume reached ∼2,000 mm^3^. The difference between different treatment groups was analysed by ANOVA with significance defined as *P*<0.05.

The above study was similarly performed in BALB/c nude mice to elucidate a role of T-cell response in PEG_2k_-Fmoc-NLG-mediated antitumour activity^21^,^25^.

### Preparation and characterizations of micelles

The drug-loaded micelles were prepared by mixing PTX (10 mM in chloroform) or DOX (10 mM in chloroform) with PEG_2k_-Fmoc-NLG(L) or PEG_2k_-Fmoc-NLG(S) (10 mM in chloroform) at various carrier/drug ratios. The solvent was removed by N_2_ flow to form a thin film of drug/carrier mixture. The film was dried under vacuum for 1 h and DPBS was added to form the drug-loaded micelles. The particle size and zeta potential of micelles were measured by a Zetasizer. The morphologies of both drug-free micelles and drug-loaded micelles were examined by TEM. The CMC was determined by using nile red as a fluorescence probe following our published protocol^34^.

### *In vitro* cytotoxicity of PTX- and DOX-loaded micelles

4T1.2 or PC3 cells at 2,000 cells/well were seeded in 96-well plates, respectively. After 12 h incubation, the cell culture medium was removed and various concentrations of free PTX, free PEG_2k_-Fmoc-NLG(L) micelles or PTX/PEG_2k_-Fmoc-NLG(L) mixed micelles were added to the cells. After 3 days of incubation, 20 μl of 3-(4, 5-dimethylthiazol-2-yl)-2,5-diphenyltetrazoliumbromide (MTT) in DPBS (5 mg ml^−1^) was added to each well and cells were further incubated for 2 h. Medium was removed and MTT formazan crystals were solubilized by 100 μl of DMSO per well. Absorbance of each well was measured with a microplate reader at wavelength of 550 nm. Untreated wells were used as controls. Cell viability was calculated as ((OD_treated_−OD_blank_)/(OD_control_−OD_blank_) × 100%). Cytotoxicity of DOX-loaded PEG_2k_-Fmoc-NLG(L) micelles was similarly examined.

### Plasma pharmacokinetics and tissue distribution

Groups of 5 female BALB/c mice were i.v. administered with TAXOL or PTX/PEG_2k_-Fmoc-NLG(L) mixed micelles at a dose of 10 mg PTX per kg. Blood samples of 50 μl were withdrawn from the retro-orbital plexus/sinus of the mice from 3 min to 12 h (3 min, 10 min, 30 min, 1 h, 2 h, 4 h, 8 h and 12 h). The blood collected in heparinized tubes was centrifuged at 2,500 r.p.m. for 15 min. To 20 μl of plasma, 350 μl of acetonitrile was added for protein precipitation and the resulting mixture was centrifuged at 12,000 r.p.m. for 5 min. Three-hundred microliters of the supernatants were collected from each sample and dried under airflow. The residues were dissolved in 50 μl of methanol and analysed by HPLC for PTX. The pharmacokinetic parameters were calculated based on a noncompartment model by Phoenix WinNonlin.

For tissue distribution study, groups of 5 BALB/c mice bearing 4T1.2 tumours of 400–600 mm^3^ were i.v. administered with PTX-loaded PEG_2k_-Fmoc-NLG (L) micelles or TAXOL at a PTX dose of 10 mg kg^−1^. Mice were sacrificed at 1 h, 2 h, 6 h, 12 h, 24 h and 48 h after injection. Major organs and tumour tissues were collected, weighed, and homogenized with 2 ml solvent (acetonitrile to H_2_O=1:1, v/v). The samples were centrifuged at 4 °C, 3,500 r.p.m. for 15 min, and the supernatants were collected and dried under airflow. The residues were then dissolved in 200 μl solvent (methanol to H_2_O=1:1, v/v) and centrifuged at 4 °C, 14,500 r.p.m. for 10 min. The supernatants were mixed with equal volume of methanol and centrifuged again at 4 °C, 14,500 r.p.m. for 10 min. Twenty microliters of the clear supernatants were injected into HPLC system for detection of PTX.

The kinetics and biodistribution of PEG_2k_-Fmoc-NLG (L) and NLG919-loaded PEG_5k_-(Fmoc-Boc)_2_ micelles were similarly performed as described above. Both released free NLG and total NLG (free NLG plus intact PEG_2k_-Fmoc-NLG (L)) were determined. Briefly, following the extraction from the blood or tissues, samples were treated with porcine liver esterases (Sigma) at a final concentration of 50 U ml^−1^. After 48 h, the total NLG (released free NLG plus NLG cleaved from PEG_2k_-Fmoc-NLG by the added esterases) was extracted twice by dichloromethane (2 × 2 ml) and dried under airflow. The samples were then similarly processed as described above and determined by a HPLC-MS system (Wastes Alliance 2695 Separation Module combined with Waters Micromass Quattro Micro TM API MS detector).

### *In vivo* antitumour activity of PTX/PEG_2k_-Fmoc-NLG(L)

*In vivo* antitumour activity of PTX formulated in PEG_2k_-Fmoc-NLG(L) micelles was similarly examined in 4T1.2 tumour model as described above. Controls included PEG_2k_-Fmoc-NLG(L), TAXOL, PTX/PEG_2k_-Fmoc-NLG(S), (PTX+NLG)/PEG_5k_-(Fmoc-Boc)_2_, oral NLG plus i.v. Abraxane, and PEG_2k_-Fmoc-NLG(L) plus Abraxane. The PTX dose was 10 mg kg^−1^ and mice received all i.v. treatments once every 3 days for 5 times. Oral NLG was given daily for 15 days. The growth of tumours was followed every three days after initiation of treatment for 19 days and relative tumour volume was calculated. The difference between different treatment groups was analysed by ANOVA with significance defined as *P*<0.05. The tumours were harvested and weighted at the end of experiment.

Similarly, a dose escalation study (5, 10 and 20 mg PTX per kg) was conducted for PTX/PEG_2k_-Fmoc-NLG(L) in 4T1.2 tumour model. The antitumour activity of PTX/PEG_2k_-Fmoc-NLG(L) was further examined in a murine melanoma model, B16, as described above.

The immune cell populations in the tumour tissues with various treatments were analysed by flow cytometry^35^. Cell suspensions from spleens or tumours were filtered and red blood cells were lysed. For extracellular staining, cells were incubated with the indicated combinations of anti- bodies (CD11b, Gr-1, CD8, CD4, CD45, F4/80 and CD206). For intracellular staining, cells were fixed and permeabilized immediately after cell surface staining according to the manufacturer's description (eBioscience), combinations of antibodies (FoxP3, IFN-γ and granzyme B) were added to cells in permeabilization buffer. For IFN-γ staining, cells were stimulated with PMA (5 ng ml^−1^) and ionomycin (500 ng ml^−1^) in presence of 10 μg ml^−1^ BFA for 4 h followed with extracellular and intracellular staining. All antibodies were purchased from BD Biosciences and flow data were collected on an LSRFortessa (BD Biosciences). The data were analysed using the FlowJo software (Tree Star Inc.).

### Statistics

All data are presented as mean±s.e.m. Differences between groups were assessed using ANOVA and *P*<0.05 was considered statistically significant.

### Data availability

The data that support the findings of this study are available from the corresponding author upon reasonable request.

## Additional information

**How to cite this article:** Chen, Y. *et al*. An immunostimulatory dual-functional nanocarrier that improves cancer immunochemotherapy. *Nat. Commun.* **7,** 13443 doi: 10.1038/ncomms13443 (2016).

**Publisher's note:** Springer Nature remains neutral with regard to jurisdictional claims in published maps and institutional affiliations.

## Supplementary Material

Supplementary Information Supplementary Figures 1-16.

## Figures and Tables

**Table 1:** Biophysical characteristics of anticancer drug-loaded PEG_2K_-Fmoc-NLG(L) micelles and blank micelles.*

**Micelles**	**Molar ratio**	**Particle size (nm)**†	**DLC (%)**
PEG_2K_-Fmoc-NLG(L)	—	96.96	—
PEG_2K_-Fmoc-NLG(L): Paclitaxel	1:1	96.57	24.7
PEG_2K_-Fmoc-NLG(L): Doxorubicin	0.5:1	97.98	30.9
PEG_2K_-Fmoc-NLG(L): Docetaxel	2.5:1	96.95	10.5
PEG_2K_-Fmoc-NLG(L): Gefitinib	2.5:1	105.1	6.1
PEG_2K_-Fmoc-NLG(L): Imatinib	2.5:1	102.1	6.7
PEG_2K_-Fmoc-NLG(L): Curcumin	2.5:1	97.50	5.1

DLC, drug loading capacity.

^*^Drug concentrations in micelle were 1 mg ml^−1^ and blank micelle concentration was 20 mg ml^−1^.

^†^Measured by dynamic light scattering sizer.

**Table 2:** IC50 of PTX or DOX in different formulations.

**Groups**	**IC50 (ng ml**^**−1**^**)**	
	**4T1.2**	**PC3**
PTX	244.56±21.05	101.30±11.25
PTX/PEG_2K_-Fmoc-NLG(L)	134.44±21.81*	88.540±6.431
DOX	268.60±27.28	548.04±58.93
DOX/PEG_2K_-Fmoc-NLG(L)	178.53±28.74	423.16±38.15

The MTT assay was performed as described for Fig. 3d,e. The IC50s were analysed by the GraphPad Prism6 software. Data are means±s.e.m. of three independent experiments. *P<0.05 (versus IC50 of PTX in 4T1.2 cells, *N*=3).

**Table 3:** Pharmacokinetic parameters of PTX in different formulations.

**Groups**	***T***_**1/2**_**(h)**	**AUC**_**0-infinity**_ **(μg h ml**^**−1**^**)**	**C**_**max**_ **(μg ml**^**−1**^**)**	**CL (ml h**^**−1**^ **kg**^**−1**^**)**	**Vd (ml kg**^**−1**^**)**
PTX/PEG_2k_-Fmoc-NLG(L)	5.552	45.23	12.98	3.242	25.97
Taxol	1.434	4.313	9.912	46.09	95.33

The experiment was performed as described for Fig. 4e. The pharmacokinetic parameters were analysed by Phoenix WinNonlin.
