# Supplementary material for: An immunostimulatory dual-functional nanocarrier that improves cancer immunochemotherapy
Source: Nat Commun. 2016 Nov 7;0:13443. doi: 10.1038/ncomms13443 (PMC5103075; doi:10.1038/ncomms13443)
Supplement: Supplementary Information — Supplementary Figures 1-16. [file ncomms13443-s1.pdf]

Supporting Information

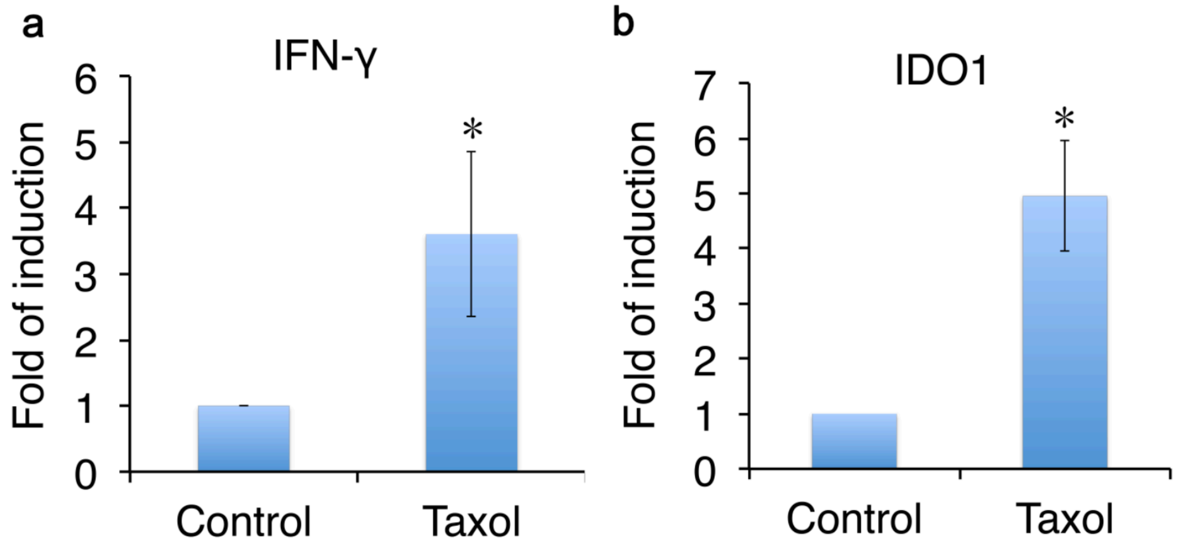

Supplementary Figure 1. RT-PCR for **(a)** IFN-γ and **(b)** IDO1 RNA transcripts in tumor tissues following Taxol treatment.

Female BALB/c mice bearing s.c. 4T1.2 tumors were treated with TAXOL i.v. once every 3 days for 5 times at a PTX dose of 10 mg kg^{-1}. Tumor tissues were harvested 6 days following the last injection. Tissues were homogenized and RNA was extracted, and RT-PCR was performed. GAPDH was used as a housekeeping gene and the relative folds of gene induction in treatment groups were calculated in comparison with PBS control group. The Bars represent means ± s.e.m. *$P < 0.05$ (vs Control, N = 3).

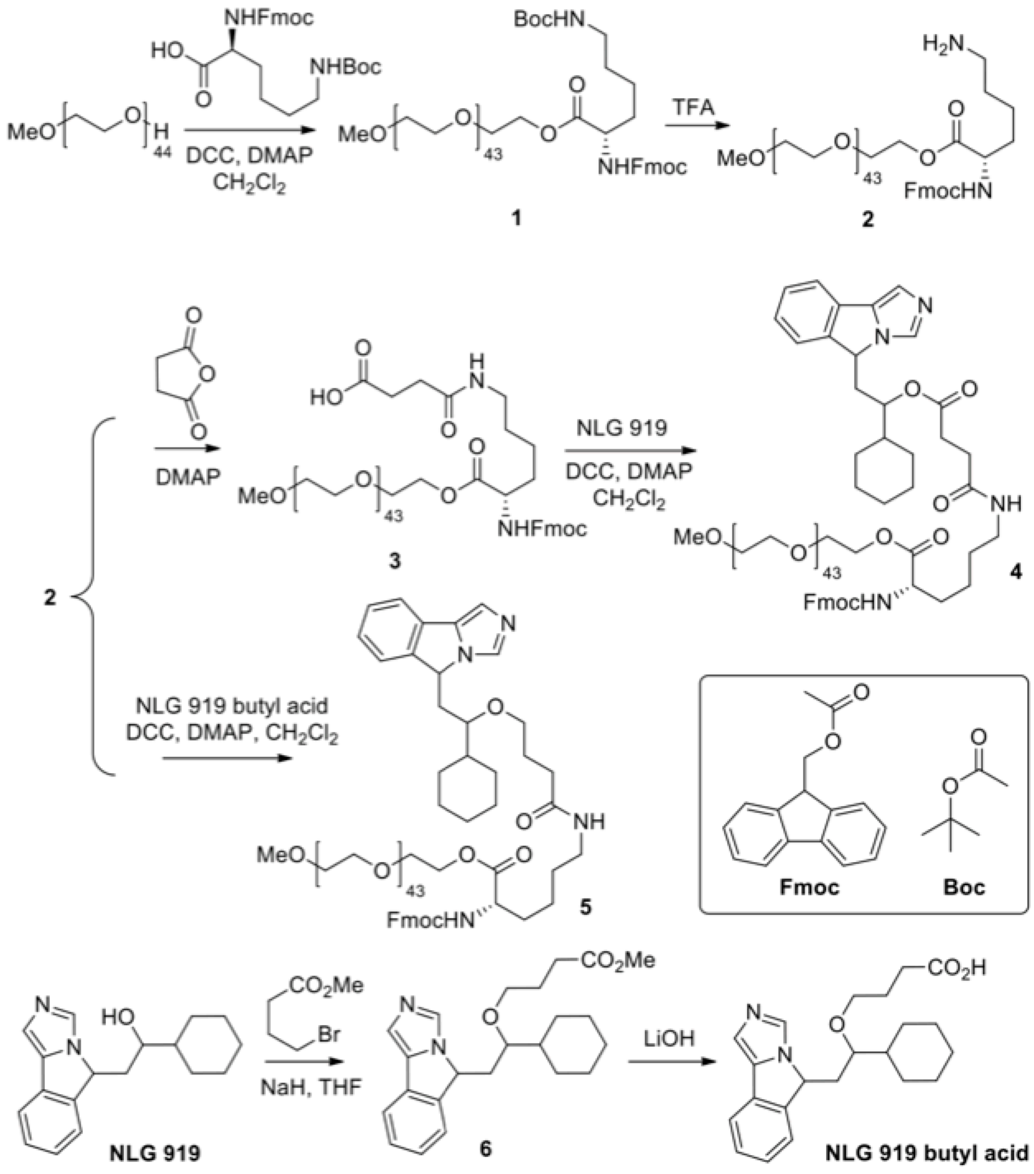

Supplementary Figure 2. Synthesis schemes of PEG$_{2k}$−Fmoc−NLG(L) (**4**) and PEG$_{2k}$−Fmoc−NLG(S) (**5**) conjugates.

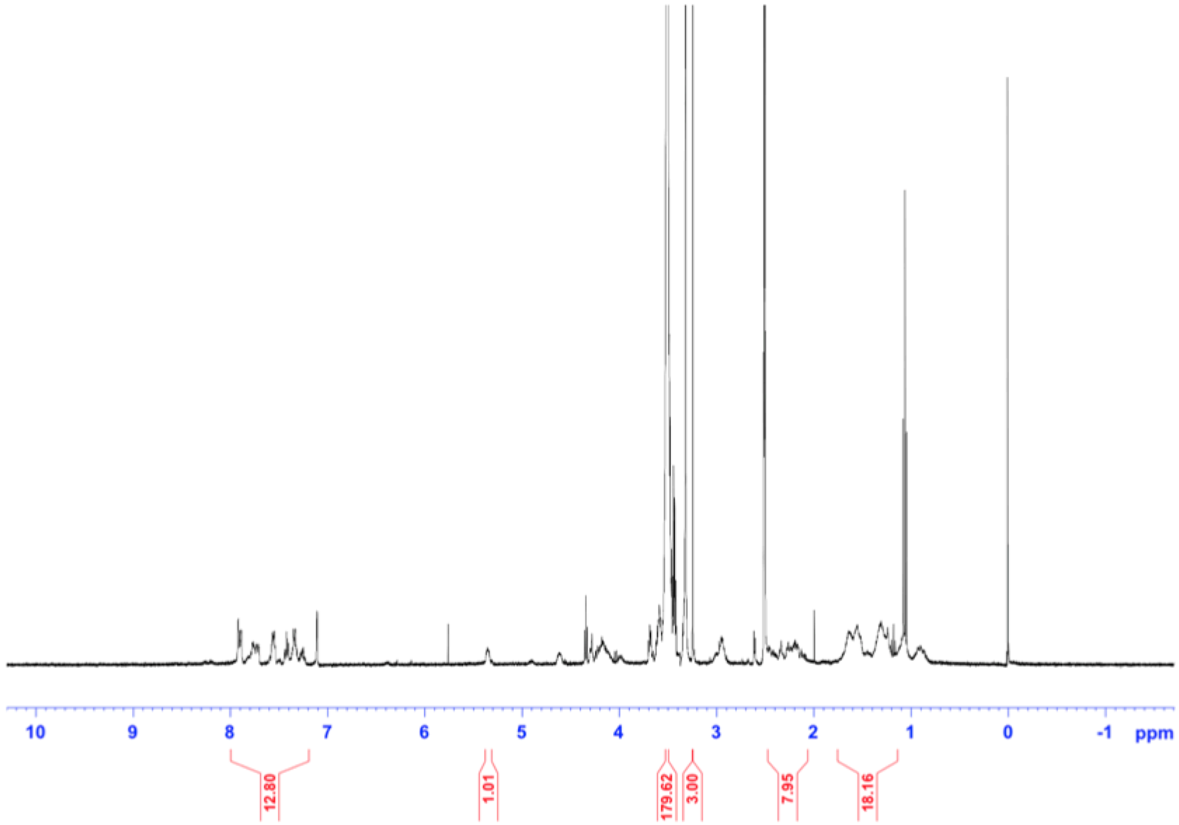

Supplementary Figure 3. ^{1}HNMR spectra (400MHz) of PEG_{2K}-Fmoc-NLG(L) conjugate in dimethyl sulfoxide-d6.

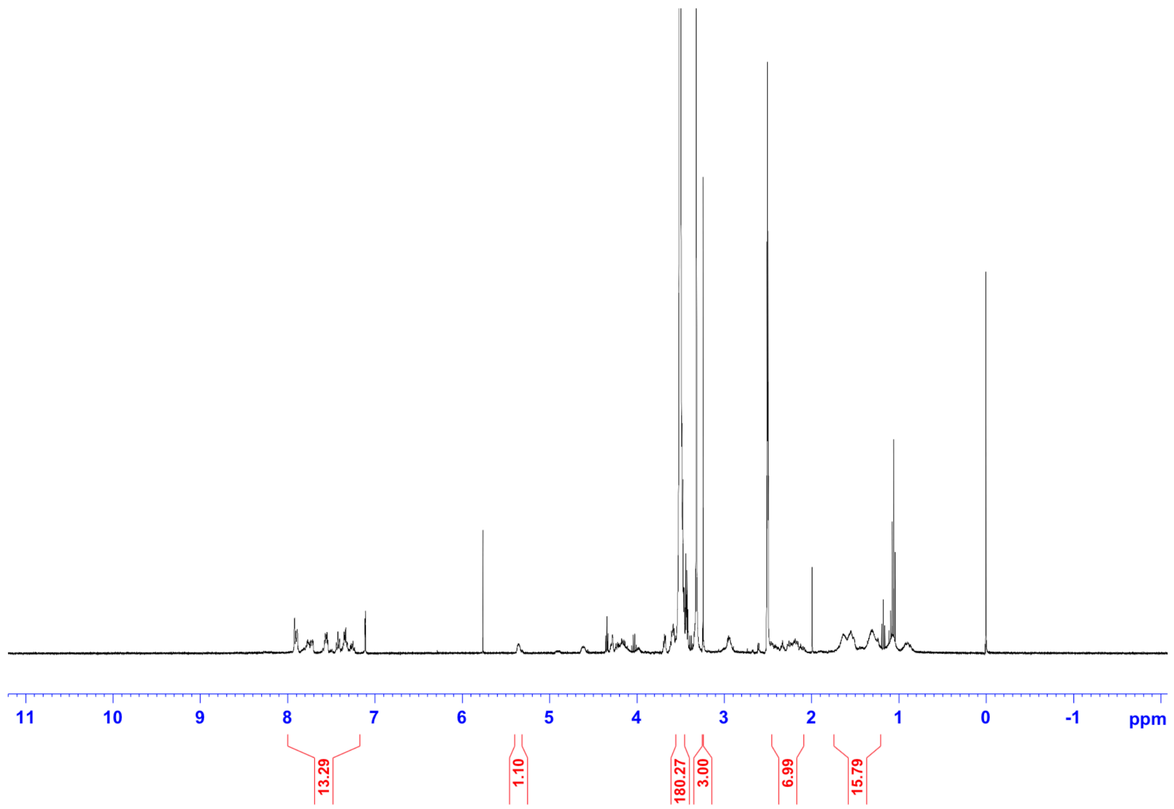

Supplementary Figure 4. ^{1}HNMR spectra(400MHz) of PEG_{2K}-Fmoc-NLG(S) conjugate in dimethyl sulfoxide-d6.

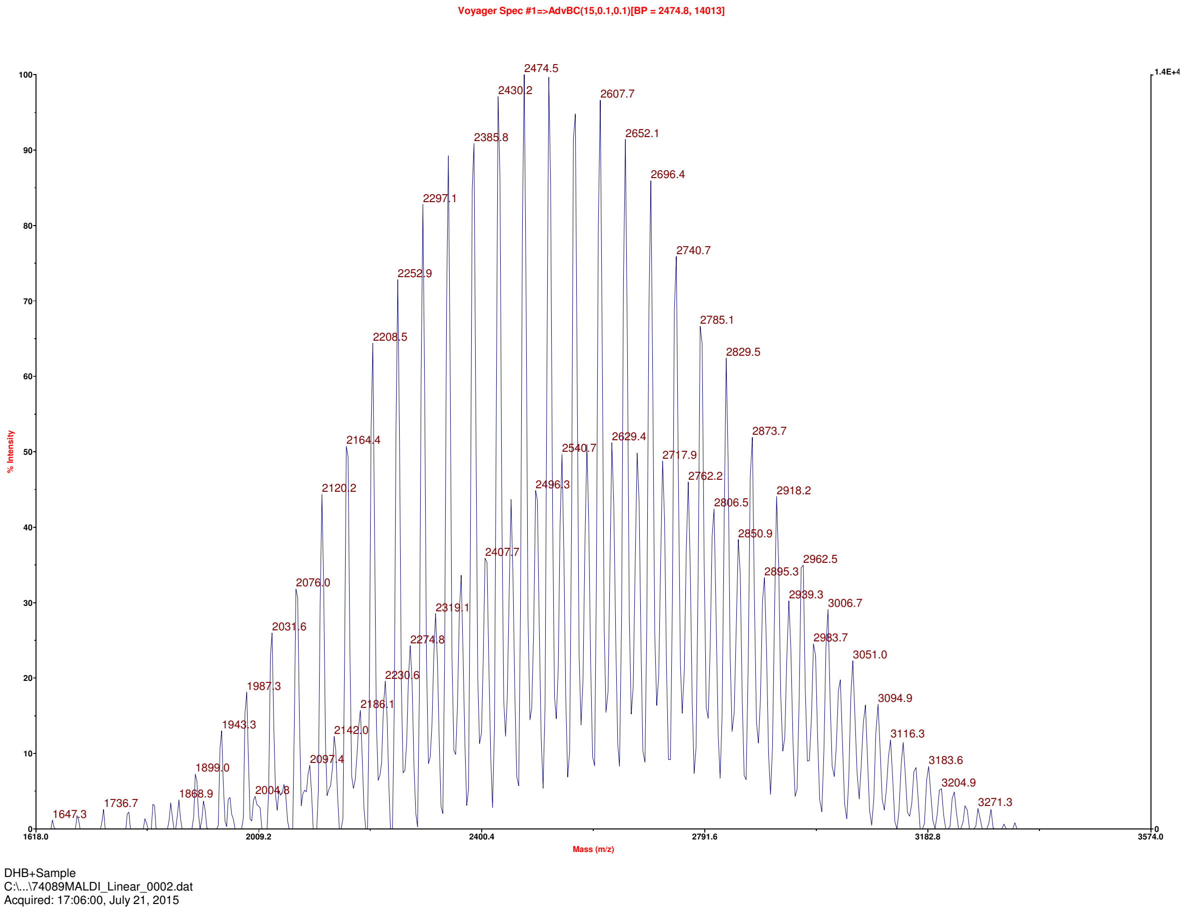

Supplementary Figure 5. MALDI-TOF of PEG_{2K}-Fmoc-NLG(L) conjugate.

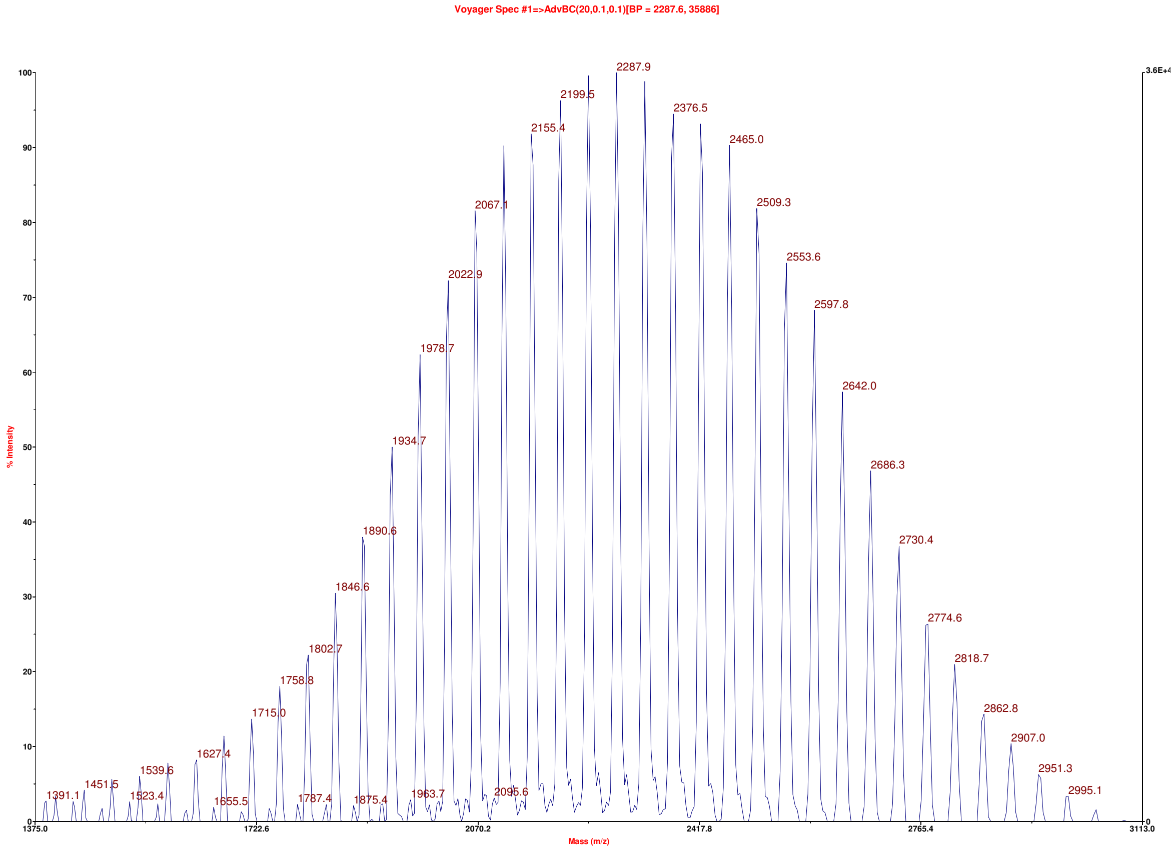

Dithranol+Sample+LiCl
C:\...\74088MALDI_Linear_0002.dat
Acquired: 17:04:00, July 21, 2015

Supplementary Figure 6. MALDI-TOF of PEG_{2K}-Fmoc-NLG(S) conjugate.

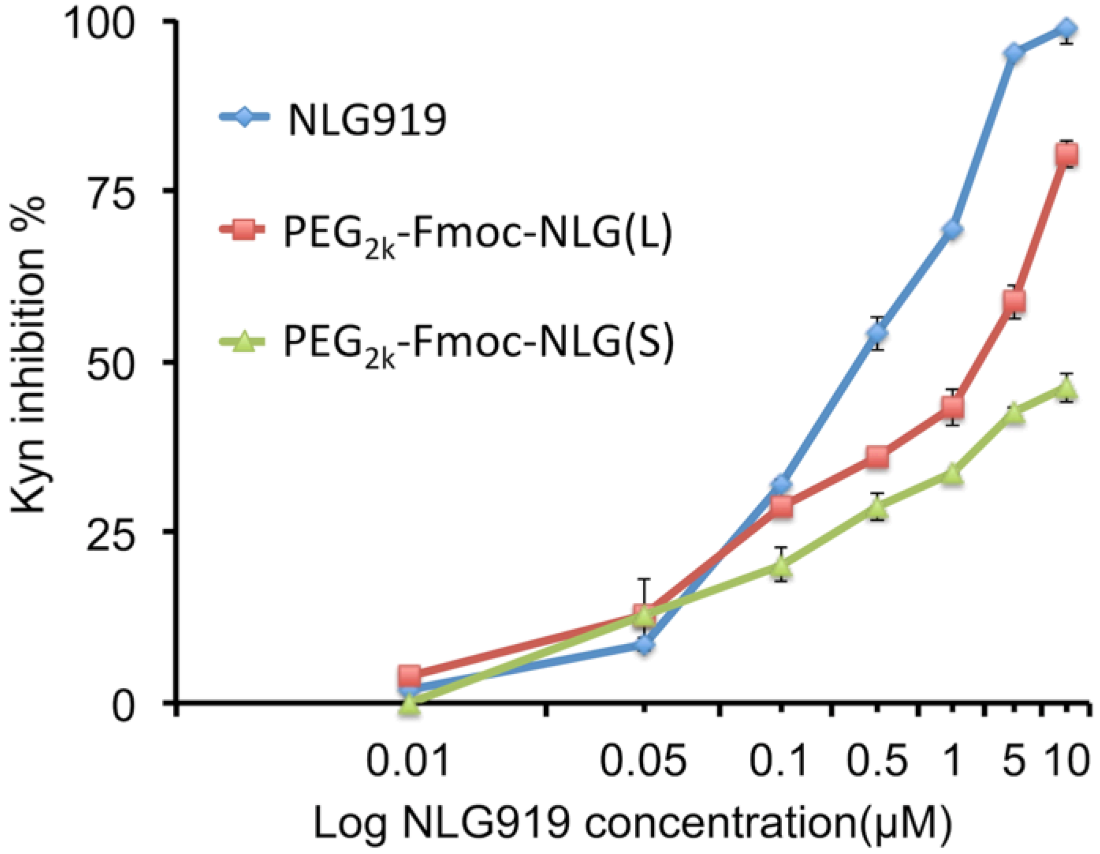

Supplementary Figure 7. PEG_{2k}-Fmoc-NLG maintains the IDO-inhibitory effect of NLG919.

HeLa cells were treated with IFN-γ (50 ng ml^{-1}) and various concentrations of PEG_{2k}-Fmoc-NLG(L), PEG_{2k}-Fmoc-NLG(S) or NLG919 for 2 days. The Kyn concentrations in the supernatants of cell culture were examined by LS-MS. Values reported are the means ± s.e.m from triplicate wells.

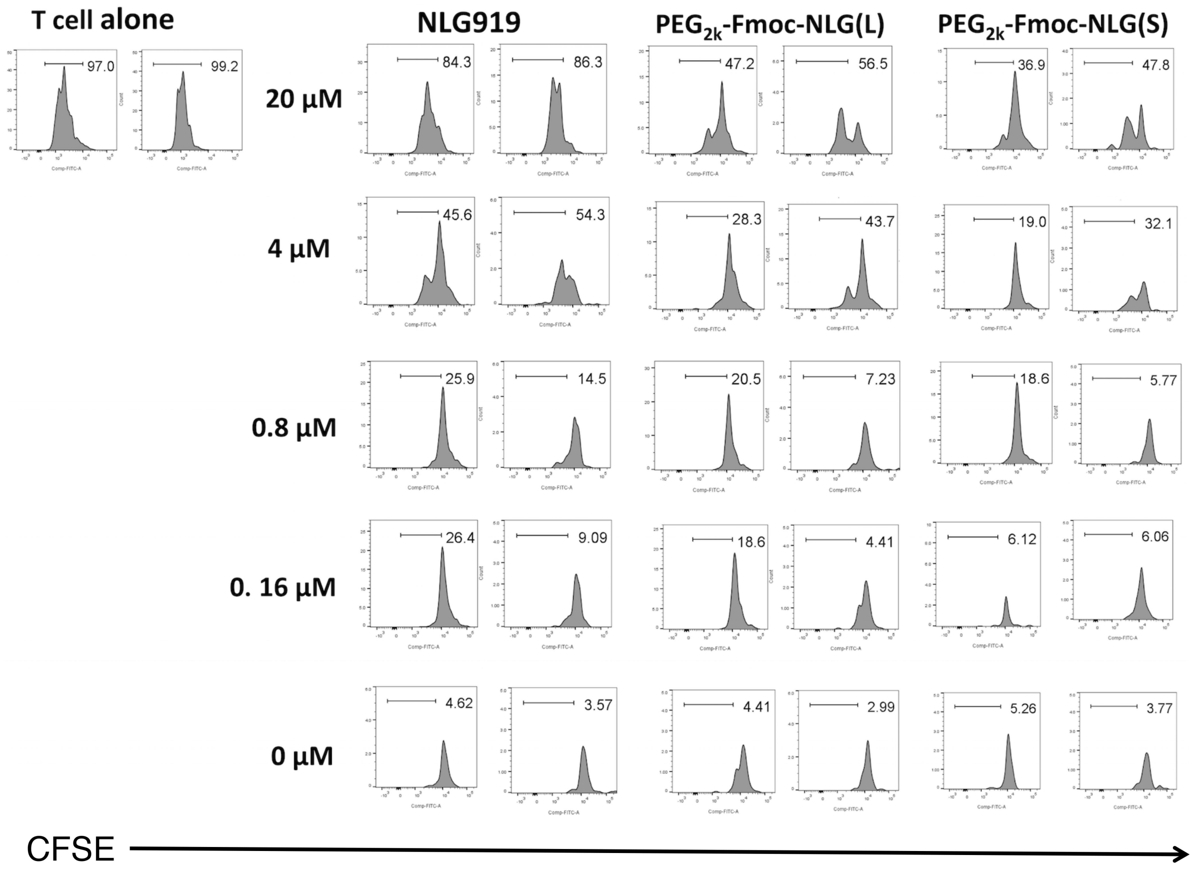

Supplementary Figure 8. Histograms of gated $CD45^{+}CD4^{+}$ and $CD45^{+}CD8^{+}$ lymphocytes.

Mixed culture of mouse splenocytes with mouse pancreatic cancer cells (Panc02) were treated with IFN-γ (50 ng ml^{-1}), IL-2 (100 U m^{-1}), and anti-CD3 antibody (100 ng ml^{-1}) for 3 days. Cells were costained for CD4 and CD8 expression. Numbers over the bracketed lines represent the percentage of the gated population that had undergone a minimum of one cell division. A representative plot of multi-color flow cytometric analysis is presented.

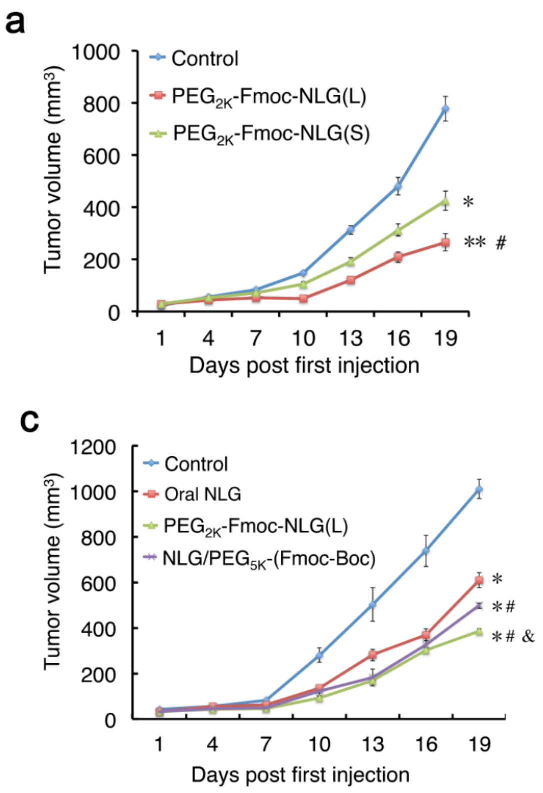

Supplementary Figure 9. *In vivo* therapeutic activities of PEG_{2k}-Fmoc-NLG. (a) PEG_{2k}-Fmoc-NLG maintained the tumor inhibitory effect. Mice bearing tumors of ~50 mm^3 received different treatments at day 1, 4, 7, 10, 13 as described in Fig. 2h. *$P < 0.05$; **$P < 0.01$ (vs control, N = 5), $^{\#}P < 0.05$ (vs PEG_{2k}-Fmoc-NLG(S), N = 5). **(b)** Lymphocyte activities were required for the *in vivo* activity of PEG_{2k}-Fmoc-NLG(L) micelles. Female BALB/c-nu/nu mice bearing 4T1.2 tumor of ~50 mm^3 were treated as described in Fig 2i. **(i)** Enhanced *in vivo* antitumor activity of PEG_{2k}-Fmoc-NLG(L) compared to oral delivery of NLG ($^{\#}P < 0.05$, N = 5) or NLG formulated in PEG_{5k}-$(Fmoc\text{-}Boc)_2$ micelles ($^{\&}P < 0.05$, N = 5), *$P < 0.05$ (vs control, N = 5). Experiment was conducted as described in Fig 2j.

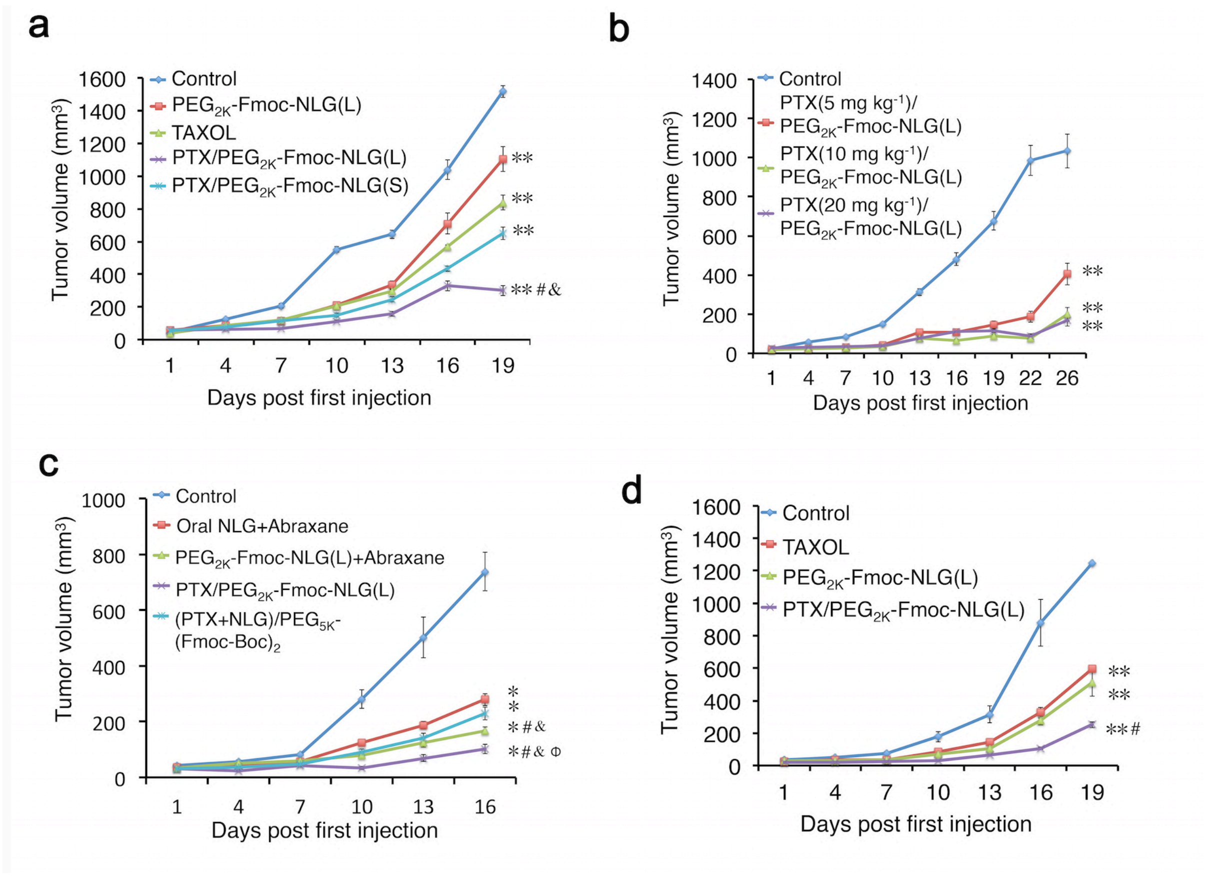

Supplementary Figure 10. *In vivo* antitumor activity of PTX-loaded PEG_{2k}-Fmoc-NLG micelles. (a) *In vivo* antitumor activity of various PTX formulations in 4T1.2 tumor model. Experiment was conducted as described in Fig. 5a. Actual tumor sizes were plotted. $^{**}P < 0.01$ (all treatment groups vs control group), $^{\#}P < 0.05$ (PTX/PEG_{2k}-Fmoc-NLG(L) vs Taxol), $^{\&}P < 0.05$ (PTX/PEG_{2k}-Fmoc-NLG(L) vs PTX/PEG_{2k}-Fmoc-NLG(S)). N= 5. **(b)** Dose-escalation study on the antitumor activity of PTX-loaded PEG_{2k}-Fmoc-NLG(L) micelles was performed as described in Fig. 5b. PTX dose was 5, 10, and 20 mg kg^{-1}, respectively. $^{**}P < 0.01$ (all treatment groups vs control), $^{\#}P < 0.05$ (20 mg PTX per kg vs 5 mg PTX per kg). N= 5. **(c)** Antitumor activity of PTX/PEG_{2k}-Fmoc-NLG(L) in a 4T1.2 tumor model in comparison to a combination of oral NLG with i.v. Abraxane, PEG_{2k}-Fmoc-NLG(L) plus Abraxane or PEG_{5k}-(Fmoc-Boc)$_2$) micelles co-loaded with PTX and NLG. Experiment was performed as described in Fig. 5c. $^{*}P < 0.01$ (all treatment groups vs control), $^{\#}P < 0.05$ (PTX/PEG_{2k}-Fmoc-NLG(L) or PEG_{2k}-Fmoc-NLG(L) + Abraxane vs oral NLG + Abraxane), $^{\&}P < 0.05$ (PTX/PEG_{2k}-Fmoc-NLG(L) or PEG_{2k}-Fmoc-NLG(L) + Abraxane vs (PTX+NLG)/PEG_{5k}-(Fmoc-Boc)$_2$), $^{\Phi}P < 0.05$ (PTX/PEG_{2k}-Fmoc-NLG(L) vs PEG_{2k}-Fmoc-NLG(L) + Abraxane), N = 5. **(d)** Antitumor activity of PTX/PEG_{2k}-Fmoc-NLG(L) in a murine melanoma (B16) model. Experiment was performed as described in Fig. 5d. PTX dose was 10 mg kg^{-1}. $^{**}P < 0.01$ (all treatment groups vs control), $^{\#}P < 0.05$ (PTX/PEG_{2k}-Fmoc-NLG(L) vs Taxol), N = 5.

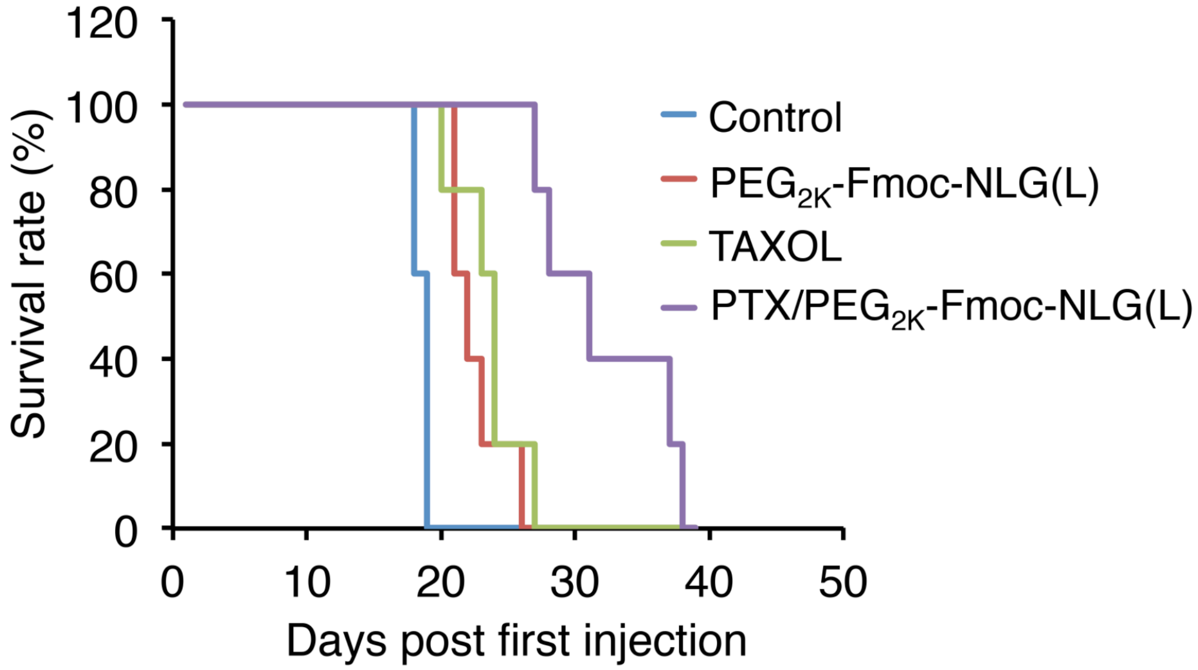

Supplementary Figure 11. Kaplan-Meier survival curves for BALB/c mice bearing 4T1.2 murine breast cancer (N=5).

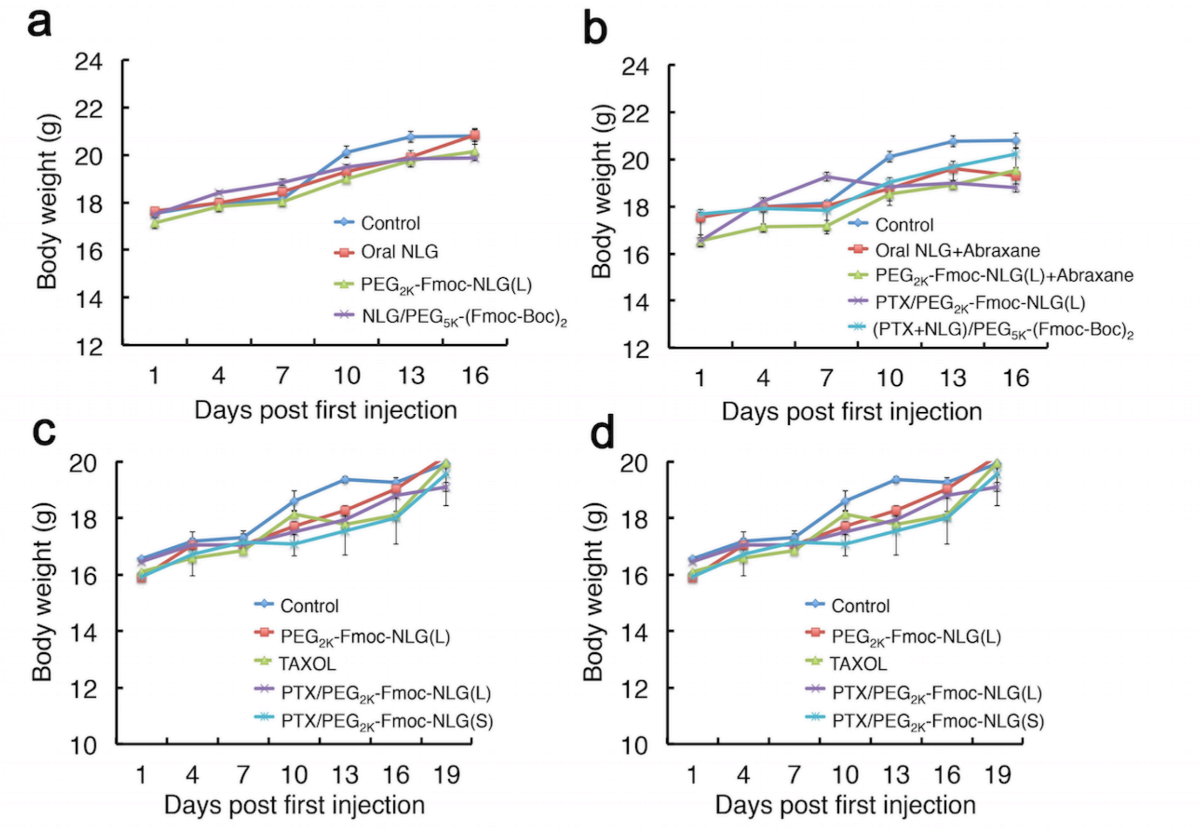

Supplementary Figure 12. Changes of body weights in mice receiving different treatments.

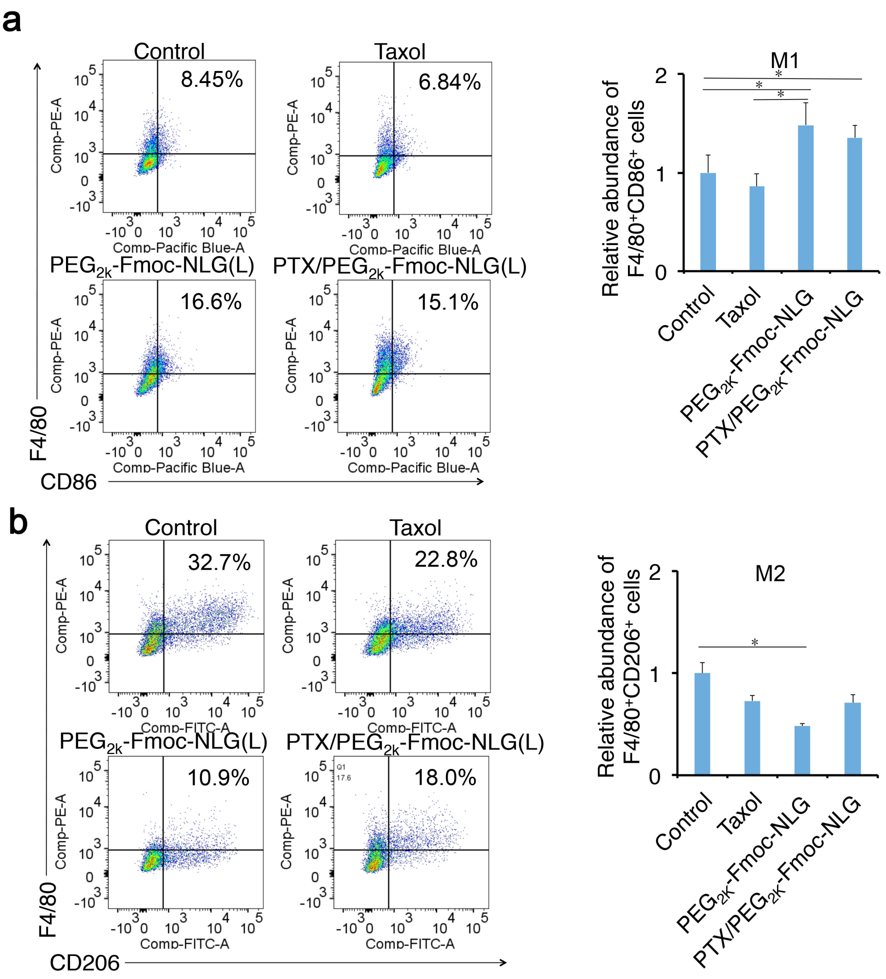

Supplementary Figure 13. Tumor-associated macrophages (TAMs) in mouse tumors. The percentages of TAM populations with specific macrophage markers (**a**) M1-type ($CD11b^+/F4/80^+/CD86^+$) and **b)** M2-type ($CD11b^+/F4/80^+/CD206^+$)) in tumor tissues were detected by flow cytometry.

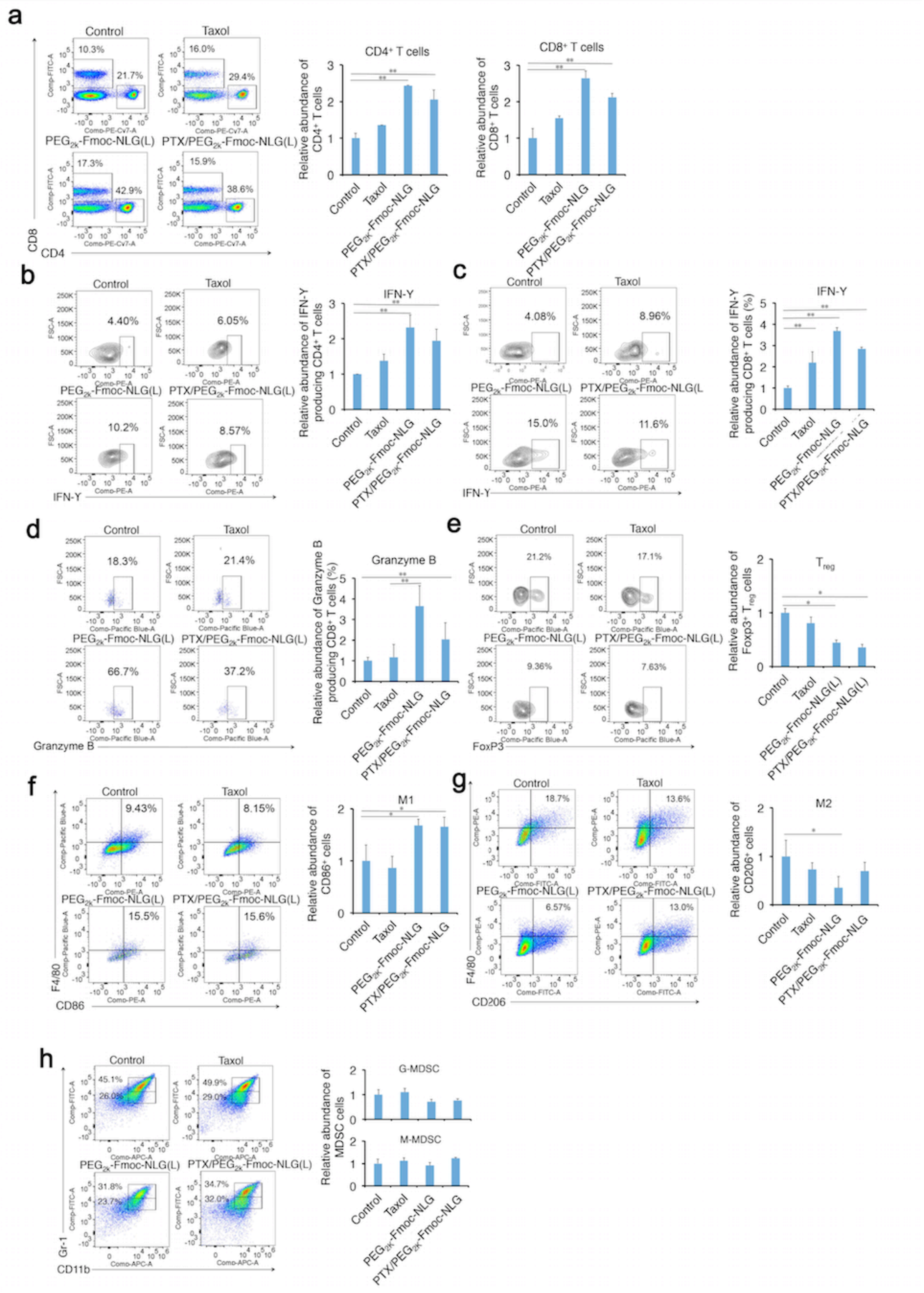

Supplementary Figure 14. Flow cytometry analysis of immune cell subsets in tumor tissues with different treatments. **(a-d)** T cell infiltration in mouse tumors treated with Taxol,

PEG_{2k}-Fmoc-NLG(L) or PTX/PEG_{2k}-Fmoc-NLG(L) at a PTX dosage of 10mg kg^{-1}. The relative abundance of $CD4^+$, $CD8^+$ **(a)**, IFN-γ positive intratumoral $CD4^+$ T cells **(b)**, IFN-γ positive intratumoral $CD8^+$ T cells **(c)**, and granzyme B-positive $CD8^+$ T cells **(d)** in tumor tissues were detected by flow cytometry. **(e)** Flow cytometry gating and histogram analysis of $FoxP3^+$ T regulatory cells in mouse tumors. **(f-g)** Tumor-associated macrophages (TAMs) in mouse tumors. The percentages of TAM populations with specific macrophage markers (M1-type ($CD11b^+$/$F4/80^+$/$CD86^+$) and M2-type ($CD11b^+$/$F4/80^+$/$CD206^+$)) in tumor tissues were detected by flow cytometry. **(h)** Flow cytometry gating and histograms analysis of $CD11b^+$/$Gr\text{-}1^+$ MDSC cells in mouse tumors. Double positive cells contain two populations, including $Gr\text{-}1^{high}CD11b^+$ granulocytic (G-MDSC) and $Gr\text{-}1^{int}CD11b^+$ monocytic (M-MDSC) MDSC subsets. The Bars represent means ± s.e.m. (*p<0.05, **p<0.01, N = 3)

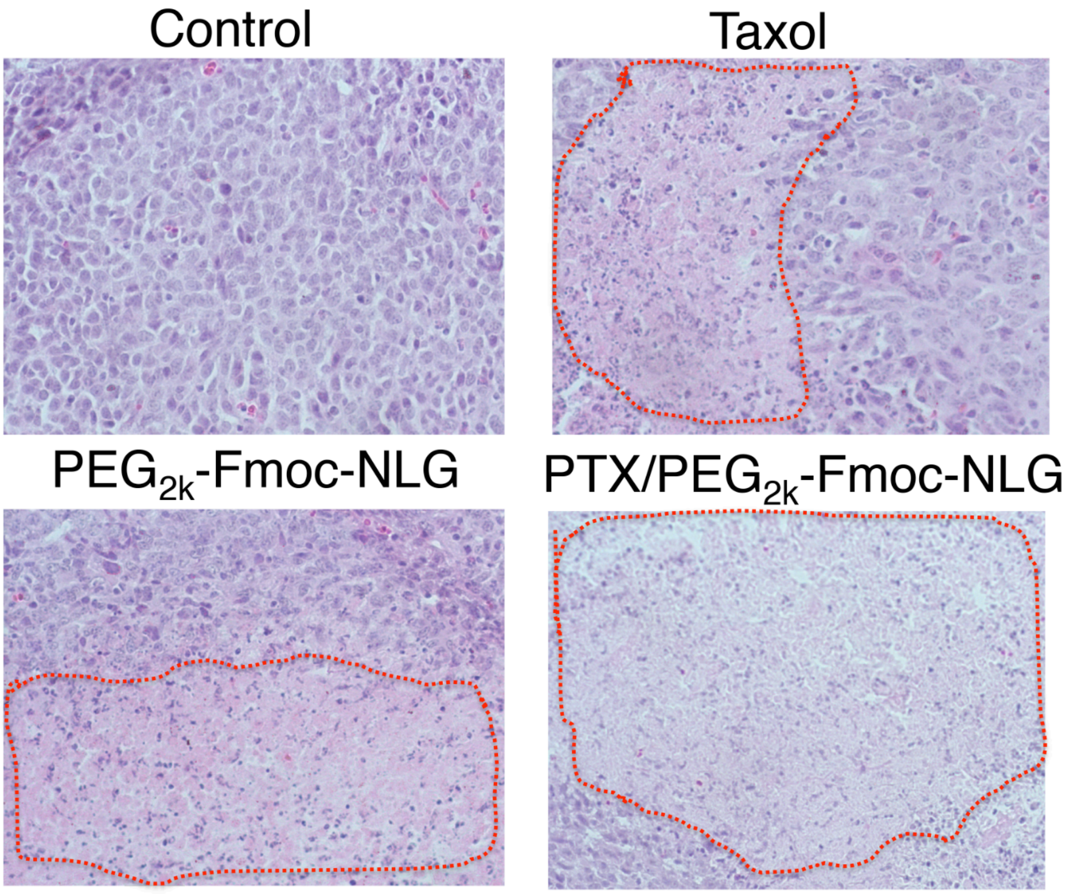

Supplementary Figure 15. H&E staining of tumor tissues.

Tumor bearing mice were treated with Taxol, PEG$_{2k}$-Fmoc-NLG(L) and PTX/PEG$_{2k}$-Fmoc-NLG(L) every 3 days for 5 times. Tumor tissues were harvested 1 day after last treatment. Nuclei and cytoplasm were stained by hematoxylin and eosin, respectively.

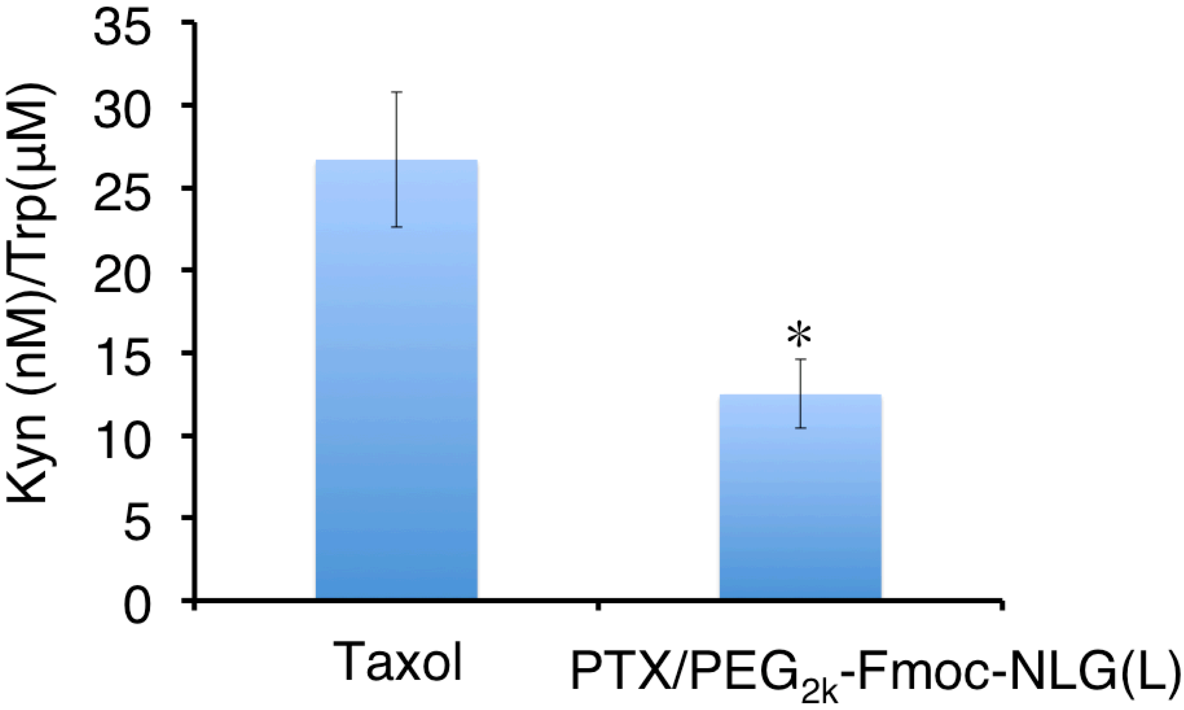

Supplementary Figure 16. PTX/PEG_{2k}-Fmoc-NLG(L) decreased kynurenine concentrations in blood in 4T1.2 tumor-bearing mice.

Female BALB/c mice bearing s.c. 4T1.2 tumors were treated with TAXOL or PTX/PEG_{2k}-Fmoc-NLG(L) i.v. once every 3 days for 5 times at a PTX dose of 10mg kg^{-1}. Blood samples were harvested 1 day after the last injection and kynurenine/tryptophan ratios were examined by LC/MS. Data are mean values ± s.e.m. of 3 experiments. *$P < 0.05$ (vs Taxol, N = 3).
